# Fecal Microbiota Transplantation in Gestating Sows and Neonatal Offspring Alters Lifetime Intestinal Microbiota and Growth in Offspring

**DOI:** 10.1128/mSystems.00134-17

**Published:** 2018-03-13

**Authors:** Ursula M. McCormack, Tânia Curião, Toby Wilkinson, Barbara U. Metzler-Zebeli, Henry Reyer, Tomas Ryan, Julia A. Calderon-Diaz, Fiona Crispie, Paul D. Cotter, Christopher J. Creevey, Gillian E. Gardiner, Peadar G. Lawlor

**Affiliations:** a Teagasc, Pig Development Department, Animal and Grassland Research and Innovation Centre, Moorepark, Fermoy, County Cork, Ireland; b Department of Science, Waterford Institute of Technology, Waterford, Ireland; c Animal and Microbial Sciences, Institute of Biological, Environmental and Rural Sciences (IBERS), Aberystwyth University, Aberystwyth, United Kingdom; d Institute of Animal Nutrition and Functional Plant Compounds, University Clinic for Swine, University of Veterinary Medicine Vienna, Vienna, Austria; e Leibeniz institute (FBN), Dummerstorf, Germany; f Department of Animal Behaviour and Welfare, Institute of Genetics and Animal Breeding, Polish Academy of Sciences, Jastrzębiec, Magdalenka, Poland; g Teagasc Food Research Centre, Moorepark, Fermoy, County Cork, Ireland; h APC Microbiome Institute, Cork, Ireland; University of California, Berkeley

**Keywords:** fecal microbiota transplantation, feed efficiency, intestinal microbiota, pigs

## Abstract

Previous studies suggest a link between intestinal microbiota and porcine feed efficiency (FE). Therefore, we investigated whether fecal microbiota transplantation (FMT) in sows and/or neonatal offspring, using inocula derived from highly feed-efficient pigs, could improve offspring FE. Pregnant sows were assigned to control or FMT treatments and the subsequent offspring to control treatment, FMT once (at birth), or FMT four times (between birth and weaning). FMT altered sow fecal and colostrum microbiota compositions and resulted in lighter offspring body weight at 70 and 155 days of age when administered to sows and/or offspring. This was accompanied by FMT-associated changes within the offspring’s intestinal microbiota, mostly in the ileum. These included transiently higher fecal bacterial diversity and load and numerous compositional differences at the phylum and genus levels (e.g., *Spirochaetes* and *Bacteroidetes* at high relative abundances and mostly members of *Clostridia*, respectively), as well as differences in the abundances of predicted bacterial pathways. In addition, intestinal morphology was negatively impacted, duodenal gene expression altered, and serum protein and cholesterol concentrations reduced due to FMT in sows and/or offspring. Taken together, the results suggest poorer absorptive capacity and intestinal health, most likely explaining the reduced body weight. An additive effect of FMT in sows and offspring also occurred for some parameters. Although these findings have negative implications for the practical use of the FMT regime used here for improving FE in pigs, they nonetheless demonstrate the enormous impact of early-life intestinal microbiota on the host phenotype.

**IMPORTANCE** Here, for the first time, we investigate FMT as a novel strategy to modulate the porcine intestinal microbiota in an attempt to improve FE in pigs. However, reprogramming the maternal and/or offspring microbiome by using fecal transplants derived from highly feed-efficient pigs did not recapitulate the highly efficient phenotype in the offspring and, in fact, had detrimental effects on lifetime growth. Although these findings may not be wholly attributable to microbiota transplantation, as antibiotic and purgative were also part of the regime in sows, similar effects were also seen in offspring, in which these interventions were not used. Nonetheless, additional work is needed to unravel the effects of each component of the FMT regime and to provide additional mechanistic insights. This may lead to the development of an FMT procedure with practical applications for the improvement of FE in pigs, which could in turn improve the profitability of pig production.

## Abstract (precis)

Here, for the first time, we investigate FMT as a novel strategy to modulate the porcine intestinal microbiota in an attempt to improve FE in pigs. However, reprogramming the maternal and/or offspring microbiome by using fecal transplants derived from highly feed-efficient pigs did not recapitulate the highly efficient phenotype in the offspring and, in fact, had detrimental effects on lifetime growth. Although these findings may not be wholly attributable to microbiota transplantation, as antibiotic and purgative were also part of the regime in sows, similar effects were also seen in offspring, in which these interventions were not used. Nonetheless, additional work is needed to unravel the effects of each component of the FMT regime and to provide additional mechanistic insights. This may lead to the development of an FMT procedure with practical applications for the improvement of FE in pigs, which could in turn improve the profitability of pig production.

## INTRODUCTION

The composition of the intestinal microbiota has been linked with growth in pigs (1–3). Moreover, recent work from our group and others has shown that microbial composition and functionality are associated with porcine feed efficiency (FE) (4–6; U. M. McCormack, T. Curiao, B. U. Metzler-Zebeli, E. Magowan, D. P. Berry, H. Reyer, M. L. Prieto, M. Harrisson, N. Rebeiz, S. G. Buzoianu, F. Crispie, P. D. Cotter, O. O’Sullivan, G. E. Gardiner, P. G. Lawlor, submitted for publication). As FE is a major determinant of profitability in pig production, strategies to improve FE are continually being sought. To date, several approaches have been applied to increase beneficial gut bacterial populations with a view to improving FE, including probiotics (7, 8), prebiotics (9–11), and synbiotics (12, 13). Here, for the first time, we attempted to improve FE through fecal microbiota transplantation (FMT) to pregnant sows and/or their offspring.

Fecal microbiota transplantation involves the transfer of donor microbiome (i.e., fecal material) to a recipient in order to establish a more desirable microbiome. The aim is to populate the gastrointestinal tract (GIT) with potentially beneficial bacteria, thereby establishing/restoring intestinal homeostasis (14, 15). To date, FMT has been used with most success to treat recurrent *Clostridium difficile* infection in humans (16). It is also under investigation for the treatment of enteric infections and inflammatory bowel disorders (14), as well as metabolic and autoimmune diseases (15). One of the main advantages of FMT is that it provides a full range of microbiota (16). However, there are limitations regarding the collection, preparation, and storage of donor feces (17), as well as the selection of suitable donors.

As regards pigs, FMT has been used to generate a human microbiota-associated pig model (18, 19) and to graft porcine microbiota into the rodent GIT (20, 21). Interestingly, FMT from three pig breeds resulted in similar intestinal structures, gene expression levels, and enzymatic activities in germfree mice as in the donor pigs (22). However, only a limited number of studies have conducted pig-to-pig microbiota transfer. In one, FMT to piglets was successful in preventing necrotizing enterocolitis in a piglet model of the disease, but there was increased neonatal mortality (23). In another study, immunologic characteristics were transferred from donor to recipient as a result of microbiota transplantation between pig breeds (24). These studies provide evidence of the ability to reprogram the porcine intestinal microbiota via FMT, with resultant alterations in host phenotype. However, the use of FMT as a tool to improve FE in pigs has yet to be investigated. Therefore, the purpose of this study was to investigate whether oral FMT with fecal extracts from highly feed-efficient pigs in sows and/or their offspring would improve FE via beneficial modulation of the intestinal microbiota.

## RESULTS

**FIG 1:**
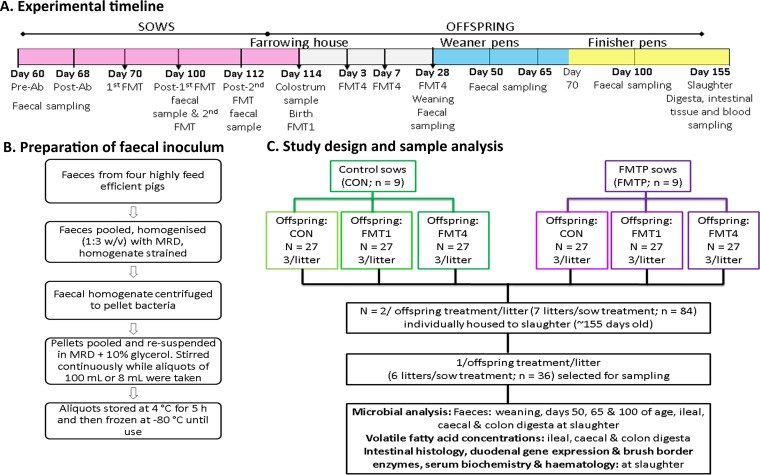
(A) Schematic of experimental timeline; details are as follows. Sow treatment groups comprised control (CON) (*n* = 9) and fecal microbiota transplantation procedure (FMTP) (*n* = 9) groups; the FMTP is described in Materials and Methods. “Pre-Ab” is preantibiotic, and “Post-Ab” is 4 days postantibiotic. On days 70 and 100, 200-ml fecal inocula were given to sows via gastric intubation. Post-first FMT fecal sampling was done 30 days after the first FMT, i.e., on day 100 of gestation. Post-second FMT fecal sampling was done 12 days after the second FMT, i.e., on day 112 of gestation. FMT1 treatment comprised a single 8-ml fecal inoculum given orally to offspring at birth, and FMT4 treatment comprised 8-ml fecal inocula given orally to offspring at birth and at 3, 7, and 28 days of age. (B) Schematic of fecal inoculum preparation; details are as follows. Pooled fecal samples were homogenized in maximum recovery diluent (MRD; Oxoid Ltd.) by stirring at 1,000 rpm for 10 min using a magnetic stirrer and then strained through a sieve to remove large particles, which were then washed through the sieve with another part of MRD. The homogenate was centrifuged for 30 min at 4,000 × *g*, and the supernatant discarded. Aliquots of the pellets in MRD + glycerol were stored at 5°C to allow microbial adjustment to low temperatures and then stored for no longer than 3 months at −80°C. (C) Schematic of study design and sample analysis.

The sow treatment by (×) offspring treatment interaction resulted in 6 treatments: control sow × control offspring (CON/CON); control sow × offspring receiving FMT once at birth (CON/FMT1); control sow × offspring receiving FMT four times, i.e., at birth and at 3, 7, and 28 days of age (CON/FMT4); FMT procedure (FMTP) sow × control offspring (FMT/CON) (FMTP refers to all steps used in the procedure for sows, i.e., antibiotic treatment, purgative, fasting, proton-pump inhibitor, and both FMTs); FMTP sow × FMT1 offspring (FMT/FMT1); and FMTP sow × FMT4 offspring (FMT/FMT4). Due to the large number of significant sow × offspring treatment interactions observed, we focused on the effect of sow or offspring treatment, and only indicate when an interaction was also observed, if relevant. While significant interactions are presented in Table 1 and several figures, all are summarized in Table S1 in the supplemental material. An experimental timeline, details of inoculum preparation, and a schematic depicting treatment assignment and sample analysis are shown in Fig. 1. All data reported in the sections discussing microbiota composition and predicted functionality are statistically significant (*P* < 0.05), and thus, *P* values are not reported in these sections.

10.1128/mSystems.00134-17.6 TABLE S1  Significant sow × offspring treatment interactions for all parameters measured in the study. Download TABLE S1, DOCX file, 0.03 MB. Copyright © 2018 McCormack et al. 2018 McCormack et al. This content is distributed under the terms of the Creative Commons Attribution 4.0 International license.

**TABLE 1:** Effects of fecal microbiota transplantation in sows and/or offspring on growth performance and carcass traits in offspring

Parameter	Value^a^ for indicated treatment in offspring^b^ of:						Pooled SEM^a^	*P* value		
	Control sows (*n* = 9)			FMTP sows (*n* = 9)^c^						
	Control	FMT1	FMT4	Control	FMT1	FMT4		Interaction	Sow	Offspring
Weight (kg)										
Birth	1.38	1.34	1.36	1.32	1.34	1.33	0.059	0.41	0.93	0.71
Weaning	8.8	8.2	8.0	7.6	7.2	7.0	2.64	0.99	0.52	0.93
70 days of age	35.2 a	29.9 b	30.4 b	29.1 bc	27.1 cd	26.4 d	2.29	0.01	0.02	0.19
155 days of age	127.9 a	116.0 c	120.9 b	115.3 c	114.2 c	111.3 c	2.87	<0.001	<0.001	<0.001

Growth performance^d^										
ADFI (g/day)	1,974	1,789	1,924	1,790	1,850	1,851	65.6	0.17	0.25	0.51
ADG (g/day)	926	850	901	873	884	870	29.7	0.31	0.51	0.56
FCE (g/g)	2.08	2.04	2.06	2.02	1.95	2.16	0.639	0.28	0.72	0.22
RFI (g/day)	21.2 a	−30.2 b	24.6 a	−30.1 b	9.4 ab	1.33 ab	21.38	0.08	0.51	0.52

Carcass traits										
Carcass wt (kg)	97.0	89.1	92.8	89.1	86.2	90.9	2.85	0.54	0.07	0.14
Kill out yield (%)	76.7	76.5	76.5	76.4	76.2	76.4	0.29	0.88	0.39	0.80
Fat (mm)	16.3	15.7	15.1	14.8	14.9	15.9	0.73	0.29	0.41	0.93
Muscle depth (mm)	52.3	50.5	50.4	52.1	51.8	53.0	1.53	0.64	0.33	0.78
Lean meat yield (%)	54.3	54.4	54.9	55.3	55.2	54.5	0.63	0.54	0.36	0.97

a Least-squares means and pooled standard errors of the means are presented. Data are from 74 pigs. At the sow treatment level, *n* = 39 offspring from control sows, and *n* = 35 offspring from FMTP sows. At the offspring treatment level, *n* = 24 in the control group, *n* = 25 in the FMT1 group, and *n* = 25 in the FMT4 group. Values within a row that do not share a lowercase letter (a, b, c) are significantly different (*P* ≤ 0.05), and values within a row that do not share a small capital letter (a, b, c) tend to be different (*P* ≤ 0.10).

b Piglets were assigned to one of three treatment groups at birth: control, FMT1 (FMT once at birth), and FMT4 (FMT at birth and at 3, 7, and 28 days of age).

c FMTP sows received FMT via gastric intubation on days 70 and 100 of gestation.

d Values shown are for growth performance from weaning to 155 days of age. ADFI, average daily feed intake; ADG, average daily gain; FCE, feed conversion efficiency; RFI, residual feed intake, calculated between day 28 and 155 days of age using a least-squares multiple-regression model of ADFI on ADG, metabolic live weight, sex, and all relevant two-way interactions, as well as the effects of back fat and muscle depth, which were recorded at slaughter.

### Impact of fecal microbiota transplantation on lifetime growth performance of offspring.

The growth performance (average daily feed intake [ADFI], average daily gain [ADG], feed conversion efficiency [FCE], and residual feed intake [RFI]) and carcass traits of offspring are presented in Table 1. Offspring from FMTP sows were 4.3 kg lighter than offspring from CON sows at 70 days of age (*P* < 0.05) and 8 kg lighter at ~155 days of age (*P* < 0.001). Moreover, FMT-treated offspring were 5.5 kg lighter than control offspring at ~155 days (*P* < 0.05). The carcass weight tended to be lighter for offspring from FMTP sows than for offspring from CON sows (*P* = 0.07). No other growth or carcass parameters examined were significantly affected by either sow or offspring treatment. However, CON/FMT1 and FMTP/CON pigs tended to have lower RFI values (calculated between weaning and slaughter) than CON/CON and CON/FMT4 offspring (*P* = 0.08).

### Analysis of fecal extracts used as inocula.

Although the data have their limitations, as they do not necessarily correlate with the abundance of live bacteria, no differences in DNA-based abundance were observed between any of the four donor fecal samples or in the inoculum pre- and postfreeze (*P* > 0.05) (Fig. S1). The relative abundances of bacterial phyla (*n* = 13) and genera (*n* = 54) in the donor feces, in samples taken during aliquoting, and in the thawed inocula are shown in Fig. S1. The inoculum samples had microbial compositions similar to those of the donor feces (Fig. S1), with members of *Firmicutes* ranging from 29.0 to 37.9% relative abundance, followed by *Spirochaetes* (21.4 to 37.1%) and *Bacteroidetes* (14.1 to 23.5%). At lower relative abundances, *Chlamydiae* (2.9 to 12.7%) and *Proteobacteria* (2.8 to 8.9%) were also observed. Genera categorized as unclassified were the most abundant (21.4 to 34.8%), followed by *Treponema* (11.3 to 21.2%), *Sphaerochaeta* (3.5 to 22.7%), *Chlamydia* (2.9 to 12.7%), *Alloprevotella* (3.7 to 9.7%), and *Prevotella* (4.3 to 5.9%).

10.1128/mSystems.00134-17.1 FIG S1  Total bacterial loads and microbial compositions at the phylum (A) and genus (B) levels in feces from donor pigs, as well as at the start and end of aliquoting of the resultant fecal extracts used as inocula and in the thawed inocula administered to sows and offspring. 1, unclassified (taxonomic assignment cutoff was set at >80%); 2, donor feces were from low residual feed intake (RFI) pigs at 130 days of age, and mean values across inoculum preparation days are presented; 3, mean values across inoculum preparation days; 4, mean values from thawed inocula administered; 5, I.S., *incertae sedis*. Download FIG S1, PDF file, 0.3 MB. Copyright © 2018 McCormack et al. 2018 McCormack et al. This content is distributed under the terms of the Creative Commons Attribution 4.0 International license.

### Influence of the FMT procedure on the microbiota of gestating sows.

No differences in bacterial loads in the baseline fecal samples collected prior to antibiotic administration were observed between sow treatments. However, following antibiotic treatment, the fecal bacterial load was 0.5 log_10_ copies of 16S rRNA gene/ng DNA lower in FMTP sows (*P* < 0.05) (Fig. S2). Thereafter, the total bacterial load was restored due to FMT, as it was higher in post-first FMT than in postantibiotic fecal samples (*P* < 0.05) (Fig. S2). In addition, the total fecal bacterial load increased from 5.92 log_10_ copies of 16S rRNA gene/ng DNA preantibiotic to 6.27 log_10_ copies/ng post-first FMT (*P* < 0.05) (Fig. S2). The antibiotic treatment also reduced fecal microbial diversity, as all three α diversity indices measured were reduced in FMTP sows compared to the results for CON sows (*P* < 0.05) (Fig. S2). Fecal microbial diversity then returned to preantibiotic values post-FMT.

10.1128/mSystems.00134-17.2 FIG S2  Effects of antibiotic administration and fecal microbiota transplantation procedure (FMTP) in sows during gestation on total bacterial load in the feces (A), fecal and colostrum microbial diversity at the genus level (B), compositions of bacterial phyla in feces during gestation and in colostrum (C), composition of bacterial genera in feces during gestation and in colostrum (D), and compositional differences within the fecal microbiota post-second FMT and within colostrum microbiota at farrowing (E). Fecal samples were from sows in control (CON) (*n* = 6) and FMTP (*n* = 6) treatment groups. Colostrum samples were from sows in CON (*n* = 4) and FMTP (*n* = 3) treatment groups. Pre-Ab, preantibiotic; Post-Ab, 4 days postantibiotic; Post-1st FMT, 30 days after first FMT, i.e., day 100 of gestation; Post-2nd FMT, 12 days after second FMT, i.e., day 112 of gestation. *, significantly different (*P* ≤ 0.05). Post-1st FMT fecal samples were only collected from FMTP sows. (C, D) Underlined phyla are those that differed in FMTP versus CON sows due to antibiotic treatment, and phyla in boldface are those that differed post-2nd FMT. Download FIG S2, TIF file, 14.6 MB. Copyright © 2018 McCormack et al. 2018 McCormack et al. This content is distributed under the terms of the Creative Commons Attribution 4.0 International license.

In the baseline fecal samples collected preantibiotic, no bacterial phyla differed in relative abundance between CON and FMTP sows (Fig. S2), but four genera were differentially abundant: *Butyricimonas*, *Fusobacterium*, *Roseburia*, and *Schwartzia* (*P* < 0.05) (Table S2). Antibiotic administration influenced the fecal microbial composition, with six phyla differing in relative abundance in CON versus FMTP sows (*Proteobacteria*, *Lentisphaerae*, and *Fibrobacteres* were lower, and *Verrucomicrobia*, *Tenericutes*, and “*Candidatus* Saccharibacteria” were higher) (Fig. S2) and 22 genera affected (13 lower in relative abundance and 9 higher) (*P* < 0.05) (Table S2). Thereafter, FMT appeared to restore the bacterial phylum profile to one similar to that found in the baseline fecal samples of the sows (Fig. S2). Higher relative abundances of the phyla “*Candidatus* Saccharibacteria” and *Tenericutes* and of three genera (*Alistipes*, *Citrobacter*, and *Ruminococcus*2) were observed after the second FMT in FMTP than in CON sows (*P* < 0.05) (Fig. S2). However, sow fecal bacterial diversity was not affected following FMT, nor was the microbial diversity of the colostrum (*P* > 0.05) (Fig. S2). A total of 77 OTUs were identified in the colostrum samples, mainly from *Firmicutes* (ranging from 44 to 76% relative abundance), *Proteobacteria* (6 to 31%), *Bacteroidetes* (3 to 29%), and *Actinobacteria* (0.01 to 6.95%) (data not shown). No differences were detected at the phylum level (Fig. S2), but *Oribacterium* and *Anaerovibrio* had higher relative abundances in FMTP than in CON sows (*P* < 0.05) (Fig. S2).

10.1128/mSystems.00134-17.7 TABLE S2  Relative abundances (%) of bacterial genera that differed between sow treatments pre- and post-antibiotic treatment. Download TABLE S2, DOCX file, 0.02 MB. Copyright © 2018 McCormack et al. 2018 McCormack et al. This content is distributed under the terms of the Creative Commons Attribution 4.0 International license.

### Fecal microbiota transplant-associated effects on microbial diversity in offspring.

**FIG 2:**
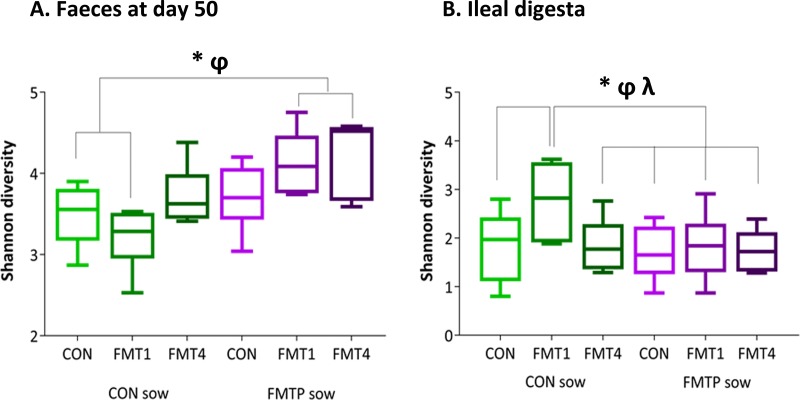
Variations in genus-level Shannon diversity of offspring microbiota in fecal samples at 50 days of age (A) and ileal digesta at ~155 days of age (B) as a result of FMT in sows and/or offspring. Data are from 36 pigs. At the sow treatment level, *n* = 18 offspring each from CON and FMTP sows. At the offspring treatment level, *n* = 12 each in CON, FMT1, and FMT4 treatment groups. *, significantly different (*P* ≤ 0.05) at the sow × offspring treatment level; φ, a sow treatment effect was seen (*P* ≤ 0.05); λ, an offspring treatment effect was seen (*P* ≤ 0.05).

Bacterial α diversity, as measured by the Shannon index, was higher in the fecal samples of 50-day-old offspring from FMTP sows than in those from CON sows (4.00 versus 3.47) but was lower in the ileum (1.75 versus 2.08) (*P* < 0.05) (Fig. 2; Table S1). In the ileum, FMT1 offspring had a higher Shannon diversity value than CON or FMT4 offspring (2.24 versus 1.77 and 1.76, respectively; *P* < 0.05).

Beta diversity was also investigated throughout the lifetime of the offspring, as illustrated by the principal component analysis (PCA) plots (Fig. S3). No differences were detected in the fecal samples collected at weaning or at 65 or 100 days of age, but at day 50, the values for FMT4 offspring clustered away from the values for CON offspring (*P* < 0.05). No differences were detected in the ileum or colon, but in the cecum, the values for offspring from FMTP sows clustered away from the values for offspring born to CON sows (*P* < 0.05).

10.1128/mSystems.00134-17.3 FIG S3  Variations in genus-level microbial diversity in offspring due to fecal microbiota transplantation (FMT) in sows and/or offspring, including the diversities in feces at 28 (A), 50 (B), 65 (C), and 100 (D) days of age and in the ileum (E), cecum (F), and colon (G), represented by principal-component analyses (PCA). The amount of variance is depicted by the percentages on each axis. *, significantly different (*P* ≤ 0.05) at the sow × offspring treatment level; φ, a sow treatment effect was seen (*P* ≤ 0.05); λ, an offspring treatment effect was seen (*P* ≤ 0.05). Data are from 36 pigs. At the sow treatment level, *n* = 18 offspring each from control and FMT procedure (FMTP) sows. At the offspring treatment level, *n* = 12 each in the control, FMT1, and FMT4 groups. Download FIG S3, PDF file, 0.4 MB. Copyright © 2018 McCormack et al. 2018 McCormack et al. This content is distributed under the terms of the Creative Commons Attribution 4.0 International license.

### Effects of FMT in sows and/or offspring on fecal/intestinal bacterial loads and compositions in offspring.

The total bacterial loads in all samples from offspring are shown in Fig. S4. The fecal bacterial load in 65-day-old offspring was higher in pigs born to FMTP sows than in those born to CON sows (6.48 versus 6.09 log_10_ copies/ng DNA) (*P* < 0.05), while in the ileum, FMT-treated offspring had a reduced bacterial load compared to that of CON offspring (3.98 versus 4.44 log_10_ copies/ng DNA) (*P* < 0.05).

10.1128/mSystems.00134-17.4 FIG S4  Effects of fecal microbiota transplantation (FMT) on bacterial loads in offspring fecal samples across all time points (A) and intestinal digesta (B). Data are from 36 pigs. At the sow treatment level, *n* = 18 offspring each from control sows and FMT procedure (FMTP) sows. At the offspring treatment level, *n* = 12 each in the control, FMT1, and FMT4 groups. *, significantly different (*P* ≤ 0.05) at the sow × offspring treatment level; φ, a sow treatment effect was seen (*P* ≤ 0.05); λ, an offspring treatment effect was seen (*P* ≤ 0.05). Download FIG S4, PDF file, 0.2 MB. Copyright © 2018 McCormack et al. 2018 McCormack et al. This content is distributed under the terms of the Creative Commons Attribution 4.0 International license.

**FIG 3:**
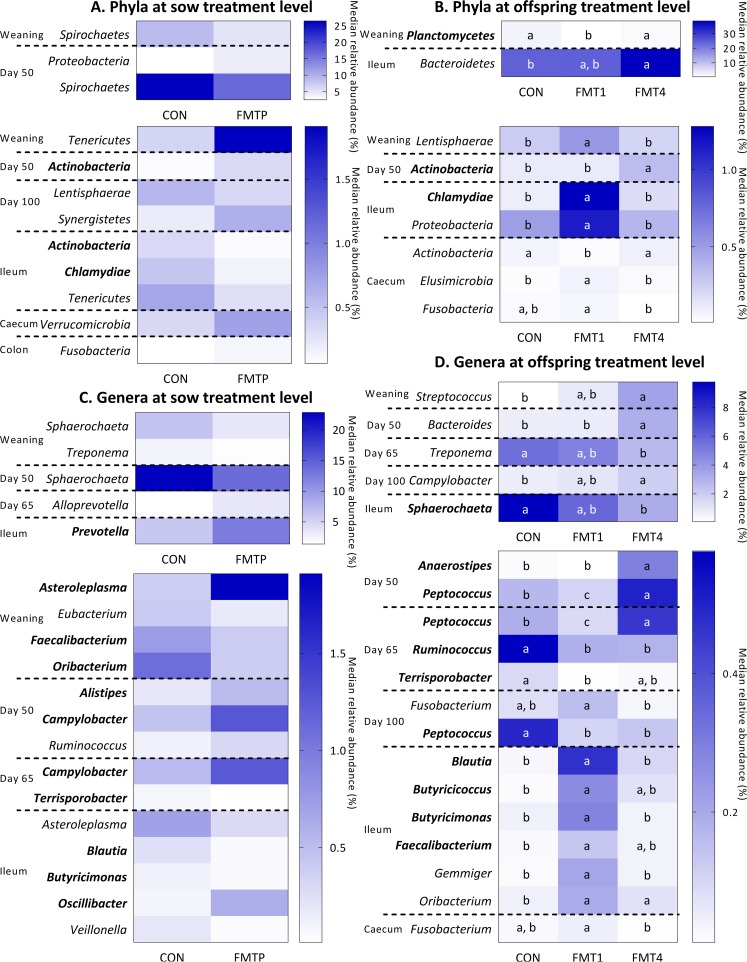
Effects of FMT in sows and/or offspring on median relative abundances (%) of bacterial phyla (A and B) and genera (C and D) in offspring fecal samples (at weaning and at 50, 65, and 100 days of age) and digesta samples (from the ileum, cecum, and colon) at the sow treatment level (A and C) and offspring treatment level (B and D). Data are from 36 pigs. At the sow treatment level, *n* = 18 offspring each from CON and FMTP sows. At the offspring treatment level, *n* = 12 each in the CON, FMT1, and FMT4 groups. Heatmaps are split by relative abundance, with more highly abundant phyla/genera shown in the upper heatmaps and less abundant taxa shown in the lower heatmaps. Phyla and genera that are in boldface are those affected by a sow treatment × offspring treatment interaction. Data for these, together with other phyla and genera also affected by a sow treatment × offspring treatment interaction, are shown in Table S1. (B and D) Within each bacterial taxon, boxes that do not share a letter (a, b, c) represent significantly different results (*P* ≤ 0.05).

Significant differences in microbial compositions of the feces and intestinal digesta at both the phylum and genus levels are shown in Fig. 3. Five bacterial phyla and 16 genera were altered due to a sow × offspring treatment interaction (Table S1). Nine of the 14 phyla detected were impacted by the FMTP in sows and 8 by FMT in offspring, and some of these, i.e., *Bacteroidetes* and *Spirochaetes*, were present at high relative abundances (>10%). Furthermore, some of the phylum effects at the sow treatment level were repeated throughout the growing period.

At weaning, offspring from FMTP sows had a higher relative abundance of *Tenericutes* (which was also higher in abundance in the feces of FMTP sows) but a lower abundance of *Spirochaetes* (Fig. 3A). *Planctomycetes* were lower but *Lentisphaerae* were higher in abundance in FMT1 offspring than in the other offspring treatments (Fig. 3B; Table S1). At 50 days of age, offspring from FMTP sows had a lower abundance of *Spirochaetes* but higher abundances of *Proteobacteria* and *Actinobacteria* (Fig. 3A). Furthermore, *Actinobacteria* were higher in abundance in FMT4-treated offspring than in CON and FMT1 offspring (Fig. 3B). At 100 days of age, the relative abundance of *Synergistetes* was higher in offspring from FMTP sows, while that of *Lentisphaerae* was lower (Fig. 3A).

In the ileum, *Tenericutes*, *Chlamydiae*, and *Actinobacteria* were less abundant in offspring from FMTP sows (Fig. 3A; Table S1). On the other hand, *Bacteroidetes* were higher in abundance in FMT4 than in CON and FMT1 offspring, while *Chlamydia* and *Proteobacteria* were higher in FMT1 offspring than in the other groups (Fig. 3B). In the cecum, *Verrucomicrobia* were enriched due to FMTP in sows (Fig. 3A). The effects of offspring treatment were more subtle; *Actinobacteria* were lower, whereas both *Fusobacteria* and *Elusimicrobia* were higher in relative abundance in FMT1 than in CON and FMT4 offspring (Fig. 3B). In the colon of offspring, FMTP in sows resulted in a slightly higher abundance of *Fusobacteria* (0.13 versus 0.06%) (Fig. 3A).

Of the 148 genera detected, 16 differed due to sow treatment and 16 due to offspring treatment (many at a relative abundance of <1%) (Fig. 3C and D). At weaning, *Sphaerochaeta* and *Treponema* (from *Spirochaetes*) and *Oribacterium* (enriched in colostrum of FMTP sows), *Faecalibacterium*, and *Eubacterium* (from *Firmicutes*/*Clostridia*) were less abundant in offspring from FMTP sows, whereas *Asteroleplasma* was enriched (Fig. 3C; Table S1). The abundance of *Sphaerochaeta* at weaning was also negatively correlated with offspring body weight at slaughter (Fig. S5). *Streptococcus* was higher in relative abundance in FMT4 offspring than in CON offspring (Fig. 3D).

10.1128/mSystems.00134-17.5 FIG S5  Effects of fecal microbiota transplantation (FMT) in sows (A) and offspring (B) on Spearman correlations between bacterial genera and offspring body weights at 155 days of age. Data are from 36 pigs. At the sow treatment level, *n* = 18 offspring each from control (CON) and FMT procedure (FMTP) sows. At the offspring treatment level, *n* = 12 each in the control, FMT1, and FMT4 groups. *, a significant correlation was seen within the treatment (*P* ≤ 0.05). Download FIG S5, PDF file, 0.2 MB. Copyright © 2018 McCormack et al. 2018 McCormack et al. This content is distributed under the terms of the Creative Commons Attribution 4.0 International license.

At 50 days of age, in agreement with the findings at weaning, *Sphaerochaeta* was less abundant in offspring feces due to FMTP in sows, whereas *Campylobacter*, *Alistipes* (also higher in abundance in the feces of FMTP sows), and *Ruminococcus* were enriched (Fig. 3C; Table S1). However, the relative abundance of *Ruminococcus* was reduced in FMT-treated offspring at 65 days of age (Fig. 3D; Table S1). *Bacteroides*, *Anaerostipes*, and *Peptococcus* were higher in relative abundance in FMT4 than in CON pigs at day 50 (Fig. 3D). Throughout the growing period, similar changes occurred; at day 65, FMT4 offspring had a higher relative abundance of *Peptococcus* than did CON offspring (Fig. 3D). *Campylobacter* was higher at day 65 in offspring from FMTP sows (Fig. 3C) and was at higher abundance at day 100 in FMT4 offspring than in their control counterparts (Fig. 3D; Table S1). On the other hand, *Peptococcus* was lower in abundance in FMT-treated offspring at day 100 (Fig. 3D; Table S1). At 65 days of age, *Alloprevotella* was higher in abundance due to FMTP in sows (Fig. 3C), whereas *Treponema* and *Ruminococcus* were lower in abundance due to FMT4 in offspring (Fig. 3D). Furthermore, *Terrisporobacter* was less abundant due to FMT treatment both in sows and offspring (Fig. 3C and D). In both the feces collected at day 100 and the cecal digesta, *Fusobacterium* was less abundant in FMT4 than in FMT1 offspring (Fig. 3D).

Most of the genus-level differences between treatments occurred in the ileum of offspring (*n* = 11) (Fig. 3C and D; Table S1). *Prevotella* and *Oscillibacter* were higher in relative abundance due to FMTP in sows, whereas *Asteroleplasma*, *Blautia*, *Butyricimonas*, and *Veillonella* were lower (Fig. 3C). The ileal *Prevotella* abundance also negatively correlated with the final body weight of offspring (Fig. S5). *Sphaerochaeta* was lower in abundance in FMT4 than in CON offspring, while *Blautia*, *Butyricimonas*, *Butyricicoccus*, *Faecalibacterium*, *Gemmiger*, and *Oribacterium* were higher in abundance due to FMT1 in offspring (Fig. 3D; Table S1). No treatment differences were observed for genera in the colon.

### Influence of FMT in sows and/or offspring on *in silico* predictions of bacterial functionality in offspring fecal/intestinal samples.

**FIG 4:**
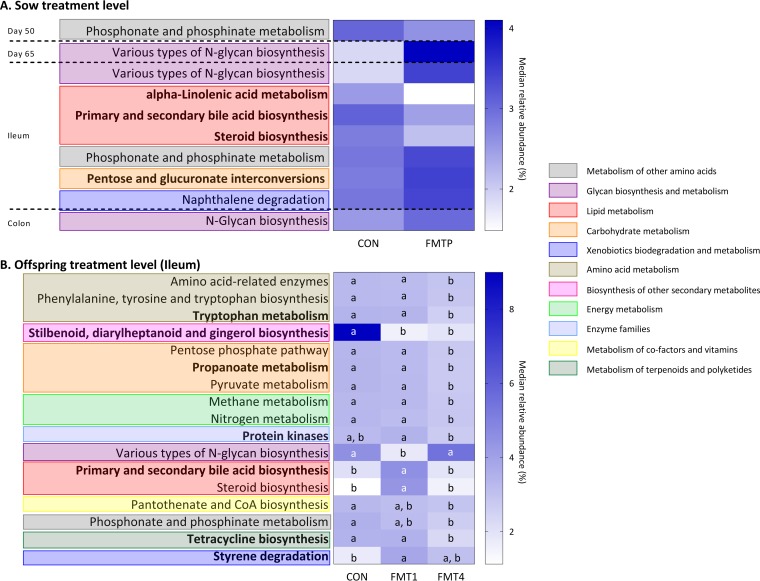
Effects of FMT in sows and/or offspring on median relative abundances (%) of predicted functional pathways in offspring fecal and intestinal microbiota at the sow treatment level (A) and offspring treatment level (B). Data are from 36 pigs. At the sow treatment level, *n* = 18 offspring each from control and FMTP sows. At the offspring treatment level, *n* = 12 each in the CON, FMT1, and FMT4 groups. Predicted pathways that are in boldface are those affected by a sow treatment × offspring treatment interaction. Data for these, together with other predicted pathways also affected by a sow treatment × offspring treatment interaction, are shown in Table S1 in the supplemental material. (B) Within each predicted bacterial pathway, boxes that do not share a letter (a, b, c) represent significantly different results (*P* ≤ 0.05).

The effects of FMT on predicted functionalities of offspring fecal and intestinal microbiota are shown in Fig. 4. Due to a sow × offspring treatment interaction, 35 predicted pathways were affected (Table S1), with 8 impacted due to FMTP in sows (Fig. 4A) and 17 due to FMT in offspring (Fig. 4B). These belonged to 11 general categories, mainly carbohydrate (*n* = 4) and amino acid and lipid (*n* = 3 each) metabolism and were mostly in the ileum (*n* = 20).

In the feces of 50-day-old offspring, those from FMTP sows had a lower predicted abundance of the phosphonate and phosphinate metabolism pathway than offspring from CON sows. The opposite occurred in the ileum of offspring from FMTP sows (Fig. 4A), but the abundance of this pathway was lower due to FMT4 in offspring (Fig. 4B). However, N-glycan biosynthesis pathways were more abundant in the feces of the offspring from FMTP sows at 50 and 65 days of age and in the colon (Fig. 4A) but were lower in the ileum of FMT1 offspring (Fig. 4B). All of the other differences in predicted microbial function observed at the sow and/or offspring treatment levels occurred in the ileum. Here, pathways relating to lipid metabolism, i.e., alpha-linolenic acid metabolism, primary and secondary bile acid biosynthesis, and steroid biosynthesis, were less abundant in offspring from FMTP sows than in those of CON sows (Fig. 4A; Table S1). However, the latter two were predicted to be more abundant in FMT1 offspring than in the other offspring treatment groups (Fig. 4B; Table S1). The naphthalene degradation pathway was enriched in offspring from FMTP sows (Fig. 4A; Table S1), and similarly, the styrene degradation pathway was higher in abundance in FMT1 offspring (Fig. 4B; Table S1). While one pathway relating to carbohydrate metabolism (pentose and glucuronate interconversions) was more abundant in offspring due to FMTP in sows (Fig. 4A; Table S1), others (propanoate, pentose phosphate, and pyruvate metabolism) were less abundant in FMT4 offspring (Fig. 4B). Similarly, pathways relating to amino acid metabolism (tryptophan metabolism, amino acid-related enzymes, and phenylalanine, tyrosine, and tryptophan biosynthesis), tetracycline biosynthesis, methane and nitrogen metabolism, protein kinases, and pantothenate and CoA biosynthesis were all predicted to be less abundant in FMT4 offspring (Fig. 4B; Table S1). Notably, the pathway predicted to be at the highest relative abundance (stilbenoid, diarylheptanoid, and gingerol biosynthesis) was less abundant in FMT-treated than in CON offspring (Fig. 4B).

### Effects of FMT in sows and/or offspring on intestinal VFA concentrations.

**FIG 5:**
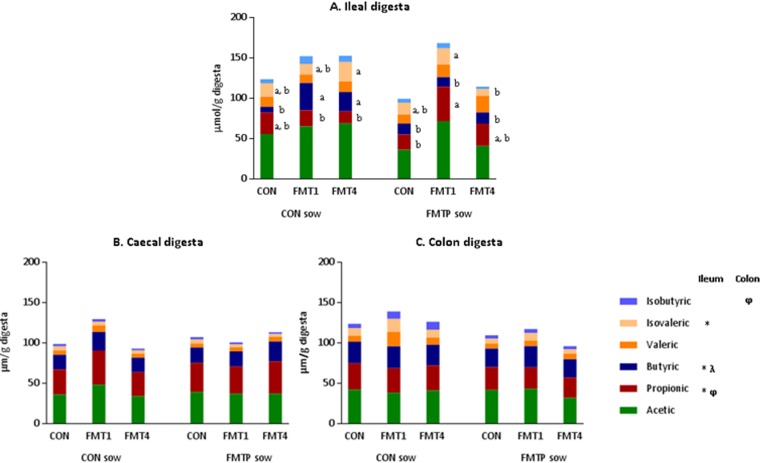
Effects of FMT on volatile fatty acid concentrations in the ileum (A), cecum (B), and colon (C) of offspring at ~155 days of age. Data are from 36 pigs. At the sow treatment level, *n* = 18 offspring each from control and FMTP sows. At the offspring treatment level, *n* = 12 each in the CON, FMT1, and FMT4 groups. For each volatile fatty acid, bars that do not share a letter (a, b, c) are significantly different due to a sow × offspring treatment interaction (*, *P* ≤ 0.05). φ indicates a difference at the sow treatment level (*P* ≤ 0.05); λ indicates a difference at the offspring treatment level (*P* ≤ 0.05).

Volatile fatty acid (VFA) concentrations were measured in the ileal, cecal, and colon digesta (Fig. 5; Table S1). In the ileum, sow × offspring treatment interactions were observed for propionic, butyric, and isovaleric acids (Fig. 5A). The ileal concentration of propionic acid was ~1.4-fold higher in offspring from FMTP sows and butyric acid was ~2-fold higher in FMT-treated offspring than in their control counterparts (*P* < 0.05). No differences were found in the cecum, but FMTP in sows caused a 1.8-fold reduction in the concentration of isobutyric acid in the colon digesta of offspring (*P* < 0.05).

### Effects of FMT in sows and/or offspring on intestinal histology in offspring.

**FIG 6:**
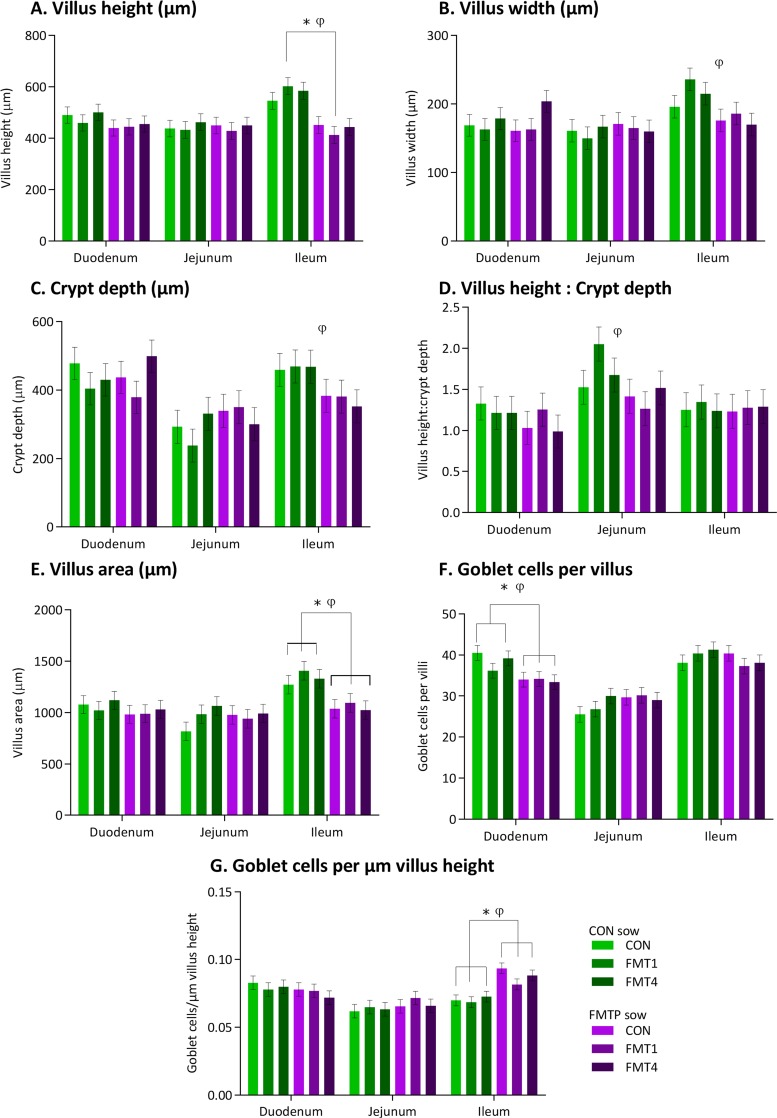
Effects of FMT in sows and/or offspring on intestinal histology in offspring, including villus height (A), villus width (B), crypt depth (C), villus height/crypt depth ratio (D), villus area (E), number of goblet cells per villus (F), and number of goblet cells per µm villus height (G). Data are from 36 pigs. At the sow treatment level, *n* = 18 offspring each from control and FMTP sows. At the offspring treatment level, *n* = 12 each in the CON, FMT1, and FMT4 groups. *, significant difference (*P* ≤ 0.05) at sow × offspring treatment level; φ, a sow treatment effect was seen (*P* ≤ 0.05).

Histological examination of tissue from the duodenum, jejunum, and ileum revealed FMT-associated differences in the offspring (Fig. 6). A sow × offspring treatment interaction was observed for the number of goblet cells per villus in the duodenum and for villus height, villus area, and number of goblet cells per µm of villus height in the ileum (Fig. 6). The number of duodenal goblet cells was lower (*P* < 0.05) (Fig. 6F) and the jejunal villus height-to-crypt depth ratio was reduced (*P* < 0.05) (Fig. 6D) due to FMTP in sows. Furthermore, the ileal villus height, width, and area were reduced (*P* < 0.05) (Fig. 6A, B, and E) and a higher number of ileal goblet cells per µm of villus height was observed in offspring from FMTP sows (*P* < 0.05) (Fig. 6G). Despite a lack of FMT-associated differences in the villus height-to-crypt depth ratio, the ileal crypt depth was reduced due to FMTP in sows (*P* < 0.05; Fig. 6C and D).

### Effects of FMT in sows and/or offspring on duodenal enzyme activity, gene expression, and blood parameters.

**FIG 7:**
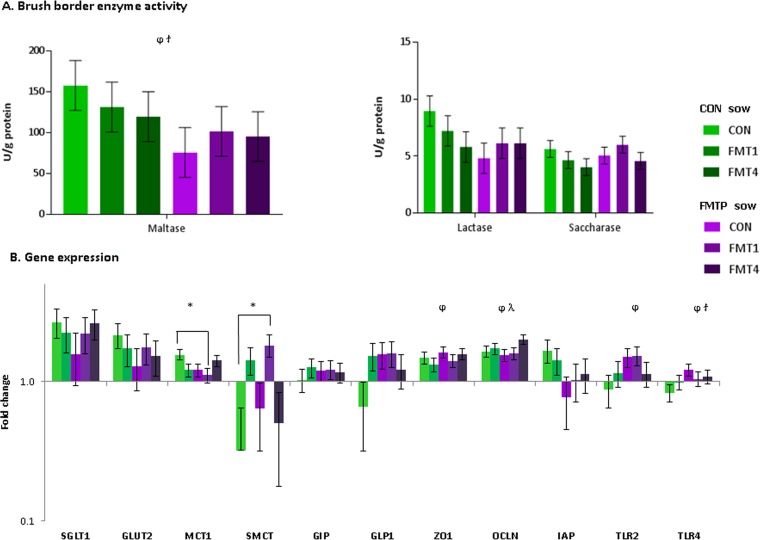
Effects of FMT in sows and/or offspring on brush border enzyme activity (A) and the expression of 11 selected genes in the duodenal mucosa (B) in offspring. Data are from 36 pigs. At the sow treatment level, *n* = 18 offspring each from control and FMTP sows. At the offspring treatment level, *n* = 12 each in the CON, FMT1, and FMT4 groups. (A and B) *, significantly different (*P* ≤ 0.05) at the sow × offspring treatment level; ϯ, tendency for differences at the sow × offspring treatment level (*P* ≤ 0.10); φ, a sow treatment effect was seen (*P* ≤ 0.05); λ, an offspring treatment effect was seen (*P* ≤ 0.05). (B) Bars represent log_10_-fold changes relative to the results for CON sow × CON offspring after normalization to glyceraldehyde-3-phosphate dehydrogenase (GAPDH), beta-actin (*ACTB*), and beta-2 microglobulin (*B2M*) gene expression levels. Error bars depict standard errors of the means. Candidate genes measured included genes encoding sodium-dependent glucose transporter 1 (*SGLT1*), monocarboxylate transporter 1 (*MCT1*), sodium-coupled monocarboxylate transporter (SMCT), intestinal alkaline phosphatase (*ALPi*), tight-junction proteins zona occludens 1 (*ZO1*) and occludin (*OCLN*), Toll-like receptor 2 (*TLR2*) and 4 (TLR4), facilitated glucose transporter member 2 (GLUT2), glucose-dependent insulinotropic peptide (*GIP*), and glucagon-like peptide-1 (*GLP1*).

The levels of disaccharidase enzyme activity and expression levels of selected genes, both measured in the duodenal mucosa of offspring, are shown in Fig. 7. For the three brush border enzymes assayed, no sow × offspring treatment interactions were observed and only maltase activity was affected, tending to be 1.5-fold lower in offspring due to FMTP in sows (*P* = 0.08) (Fig. 7A).

Regarding gene expression, a sow × offspring treatment interaction was observed for monocarboxylate transporter 1 (*MCT1*) and sodium-coupled monocarboxylate transporter (*SMCT*), and FMTP in sows resulted in upregulation of the genes encoding the tight junction protein zona occludens 1 (*ZO1*), as well as Toll-like receptor 2 (*TLR2*), in offspring compared to their expression in offspring from CON sows (*P* < 0.05) (Fig. 7B). Likewise, the gene encoding the tight junction protein occludin (*OCLN*) was upregulated in offspring due to FMT in sows or offspring (*P* < 0.05).

No interaction effect was observed for any of the serum biochemistry parameters measured at slaughter (*P* > 0.05) (Table S3), but FMTP in sows led to lower cholesterol concentrations in offspring (*P* < 0.05). On the other hand, FMT-treated offspring had lower serum total protein concentrations than control offspring (*P* < 0.05).

10.1128/mSystems.00134-17.8 TABLE S3  Effects of fecal microbiota transplantation (FMT) on hematological and serum biochemistry parameters in offspring at ~155 days of age. Download TABLE S3, DOCX file, 0.02 MB. Copyright © 2018 McCormack et al. 2018 McCormack et al. This content is distributed under the terms of the Creative Commons Attribution 4.0 International license.

The values for hematological parameters measured at slaughter are shown in Table S3. A sow × offspring treatment interaction was observed for monocyte number and red cell distribution width (*P* < 0.05). The only treatment effect observed was that offspring from FMTP sows had lower mean volumes of both corpuscles and platelets than offspring from CON sows (*P* < 0.05).

## DISCUSSION

Recent studies from our group and others have identified a link between the porcine intestinal microbiota and feed efficiency (FE) (4–6; U. M. McCormack et al., submitted). Consequently, the present study investigated, for the first time, the use of FMT as a tool to manipulate the intestinal microbiota with a view to improving FE in pigs. However, contrary to our hypothesis, transplantation of microbiota from highly feed-efficient pigs into gestating sows and/or their offspring did not consistently recapitulate the highly feed-efficient phenotype in the recipients, although some tendencies for improved FE were observed under certain treatment combinations. Instead, FMT in sows and/or their offspring resulted in a depression in offspring weight. This became apparent 70 to 100 days after the last offspring inoculation and persisted through to slaughter at ~155 days of age.

The FMTP appeared to be an effective means of repopulating the sow intestine following antibiotic treatment, allowing recovery of both bacterial load and diversity. Compositional differences were also evident within the sow fecal microbiota post-FMT, but microbial diversity was not affected. Although the fecal microbiota composition of the FMTP sows reverted to baseline at the phylum level, three genera, which did not differ at baseline (*Alistipes*, *Ruminococcus*2, and *Citrobacter*), were enriched 12 days after the second FMT. *Alistipes* has previously been suggested as FE enhancing in chickens (25), which may help to explain the FMT-associated tendencies for improvements in FE observed in certain offspring groups in the present study, and *Ruminococcus*2 plays a central role in the fermentation of complex carbohydrates (26). *Alistipes* and *Ruminococcus* (albeit not *Ruminococcus*2) were also enriched in the feces of offspring from FMTP sows, as was the *Tenericutes* phylum, which was also enhanced in the FMTP sows. This, together with the increased bacterial load and fecal diversity found in these offspring at certain time points, suggests a beneficial maternal influence, as increased intestinal microbial diversity is generally associated with improved gut health in humans (27) and reduced susceptibility to postweaning diarrhea in pigs (28). On the other hand, *Citrobacter* comprises species that are opportunistic pathogens in humans (29), although no such link has been made in pigs to date (30). This may suggest the transfer of an undesirable microbiome to offspring, which could account for the depression in offspring body weight, albeit no differences in *Citrobacter*’s relative levels of abundance were found in the offspring.

Colostrum likely acts as a vehicle for microbiota transfer to offspring (31, 32). Here, the FMTP in sows impacted the bacterial profile of colostrum, albeit to a limited extent, with only two relatively low-abundance genera affected. Furthermore, these FMTP-associated compositional differences in the colostrum were not carried through to the offspring. *Oribacterium*, for example, was more abundant in the colostrum from FMTP sows but was less abundant in the feces of pigs weaned from these sows (but more abundant in the ileum of finisher pigs treated once with FMT).

FMT in sows and/or offspring had its greatest effect in the ileum of offspring, influencing the composition and predicted functionality of microbiota, VFA concentrations, and histology. Biologically, this is of major relevance, given the importance of the ileum in nutrient absorption and immune response (33). A higher abundance of *Bacteroidetes* was observed in the ileum of offspring receiving four inoculations, and increased levels of fecal *Bacteroidetes* have previously been linked with lower adiposity in pigs (3, 34). Additionally, the increased abundance of *Bacteroides* in the 50-day-old offspring and of *Prevotella* in the ileum mediated by FMT in both sows and/or offspring is in accordance with lower adiposity in pigs and poorer FE, respectively, as previously documented (3, 5, 6), and may have also contributed to the reduction in offspring body weight found.

Butyric acid producers (including *Faecalibacterium*, *Oribacterium*, and *Eubacterium* from *Clostridiales*), linked with improved gut health and metabolism (35–37), were also impacted due to FMT in sows and/or offspring. For instance, the reduction in *Faecalibacterium* due to FMTP in sows may help to explain the FMT-associated reduction in body weight observed, as it is known for its anti-inflammatory properties and linked with heavier body weight in pigs (37, 38). Furthermore, multiple inoculations in offspring amplified the negative impact in some instances. For example, the abundances of *Streptococcus* and *Campylobacter* (some species of which are pathogenic/potentially pathogenic to pigs [39, 40]) and *Bacteroides* (linked with lower fatness, as outlined above) were higher, while *Treponema* (positively associated with porcine FE [6]) and *Sphaerochaeta* (highly enriched in fermentation and carbohydrate metabolism genes [41]) were less abundant in offspring that received FMT four times than in offspring that received FMT only once.

The FMTP in sows and FMT once or four times in offspring also had additive effects on the relative abundance of certain taxa present within the microbiomes of offspring. This was the case for the higher abundance of *Campylobacter* in the feces of 50- and 65-day-old offspring receiving FMT once from FMTP sows than in the feces from offspring receiving the same treatment but from control sows, which suggests that the combination of maternal and offspring FMT may be detrimental, given that some *Campylobacter* species are potentially pathogenic to pigs (39). Conversely, the lower abundance of *Terrisporobacter* in 65-day-old pigs due to the combination of FMTP in sows and FMT in offspring (either once or four times) may be considered beneficial, given that members of *Terrisporobacter* have been associated with infection in humans (42). The combinative effect of maternal and offspring FMT was also evident as regards phenotypic effects, with a much lower slaughter weight and a greater impact on ileal villus height seen in pigs on the combined treatments than in FMT-treated offspring from control sows.

In addition to reprogramming microbiota composition, it is notable that a number of health-related predicted microbial pathways, such as those associated with lipid metabolism and biosynthesis of other secondary metabolites (stilbenoid, diarylheptanoid, and gingerol biosynthesis), were reduced in the ileum of offspring due to FMT. More reductions in predicted lipid metabolism pathways were observed in pigs receiving FMT four times, and for one of these pathways (α-linolenic acid metabolism), there was also an additive effect of the maternal FMT regime in combination with offspring inoculation. Some of these pathways have previously been shown to have anti-inflammatory effects (43–45) and could be associated with pig growth, as the stilbenoid, diarylheptanoid, and gingerol biosynthesis pathway, for example, was enriched in high-body-weight rabbits (46). In contrast, pathways related to xenobiotic biodegradation and N-glycan biosynthesis were increased in abundance due to FMT, and the latter may be related to the higher abundance of *Campylobacter* found in these pigs. A similar link was previously made in rabbits (46), as the N-glycan biosynthesis pathway was first identified in *Campylobacter jejuni* (47, 48).

It is difficult to attribute the FMT-associated differences in VFA production to a particular phylotype. However, our data show an increase in propionic acid producers (e.g., *Ruminococcus*) (49) in the ileum due to maternal FMT, which likely accounts for the higher ileal propionic acid concentrations in these animals. Similarly, several butyric acid producers (*Butyricicoccus*, *Gemmiger*, and *Faecalibacterium*) were enriched due to FMT in offspring, along with concomitant increases in ileal butyric acid concentrations. Butyric acid is generally associated with improved gut health (50, 51). However, due to its role in regulating cytokine production (52), one could speculate that there may have been FMT-associated activation of the immune system and, consequently, diversion of energy away from weight gain. Furthermore, the increased duodenal expression of *TLR2* in offspring from FMTP sows suggests increased innate host defense signaling triggered by the altered intestinal microbiota (53, 54). Interestingly, the levels of *TLR4* and *TLR8* gene expression were previously increased when acute colitis was induced in pigs that had received a microbiota transplant from one pig breed but not another (24).

Although there is limited information to relate the intestinal microbiota to intestinal structure and function, increased abundances of members of *Clostridia* have been correlated with increased ileal villus height in pigs (55). This is in agreement with our findings, as we found the inverse due to FMTP in sows, i.e., a lower abundance of genera within the *Clostridia* class (e.g., *Blautia*, *Faecalibacterium*, *Butyricicoccus*, and *Ruminococcus*) and reduced villus height. Interestingly, an FMT-associated upregulation of *OCLN* and *ZO1* genes, encoding tight junction proteins involved in mucosal barrier function (56, 57), was observed, suggesting a more selective duodenal paracellular permeability and, thus, impaired absorptive capacity. Additionally, the greater number of goblet cells may result in overproduction of mucin in the ileum, also contributing to an overenhanced barrier function, a possible response to the inoculated microbes. Increased mucin secretion may also result in decreased nutrient absorption, as excess mucin acts as a physical barrier (58). In fact, previous work from our group showed that less-feed-efficient pigs had more ileal goblet cells (4). Similarly, the FMT-associated reductions in ileal villus height and area most likely lead to decreased nutrient absorption (59). Taken together, these findings help to explain the FMT-associated reduction in offspring body weight. Regarding metabolic effects, FMT was associated with reduced serum protein and cholesterol concentrations, which could have been a direct consequence of either decreased intestinal absorption or a microbiota-associated reduction in the relative abundances of predicted lipid metabolism pathways. Interestingly, the maternal FMTP resulted in higher ileal propionic acid concentrations in offspring, and propionate has the potential to lower blood cholesterol, as found in rats (60).

The negative impact of FMT observed in the present study could be attributed to the inoculum used. It comprised *Firmicutes*, *Bacteroidetes*, and *Proteobacteria*, identified as core phyla in the pig intestine (61). However, *Spirochaetes* and *Chlamydiae*, members of which are pathogenic to pigs (62), were both higher in relative abundance in the donor feces than is usually the case for finisher pigs (63), and this was also found in the inoculum. However, this was not reflected in the feces of sows receiving FMT, with these phyla actually less abundant in these animals than in the controls, although the FMT treatment at birth in the offspring did increase *Chlamydiae* abundance in the ileum later in life. There is also the question of donor suitability, as the donors were finisher pigs (~130 days of age) and the recipients were adult sows and neonatal piglets (the latter with a naive/essentially nonexistent microbiome). The issue of nonideal donors has been highlighted recently in humans, when a woman developed new-onset obesity after FMT from a healthy but overweight donor (64). Furthermore, there is always a concern that pathogens can be transferred to the recipients (65), and the donor pigs used in the present study, although from a high-health-status herd and highly feed efficient, were not screened for pathogens.

It could also be argued that the negative effects observed in the present study were due to the antibiotic and/or purgative administered to sows as part of the FMT regime. It is well known that antibiotics perturb the porcine intestinal microbiota (66, 67), and similar effects were found here. Laxatives also alter the intestinal microbiota composition, at least in humans (68). Both interventions may also have altered the intestinal mucus layer, thereby facilitating intestinal damage by transplanted commensals. However, neither antibiotics nor the purgative was administered to offspring, and similar FMT-associated effects, both microbiological and physiological, occurred in these animals.

### Conclusions.

In conclusion, FMT had a greater impact on offspring than on sow microbiota, irrespective of whether it was delivered directly or via maternal inoculation. This is most likely a reflection of the fact that it is easier to influence a naive than an adult microbiome. Regardless, the FMTP in sows during late gestation and/or FMT in neonatal offspring had a detrimental effect on the lifetime growth of offspring. In general, bacteria (and their predicted pathways) with a role in nutrient utilization and intestinal health, some with a positive association with porcine FE, were less abundant as a result of FMT. This may relate to altered physiological parameters, as intestinal morphology was negatively impacted and intestinal permeability- and VFA transport-related mucosal gene expression levels were altered. Together, these data suggest reduced nutrient absorptive capacity and a less metabolically efficient microbiome, possibly explaining the reduced weight at slaughter. Some of the effects were also additive, with FMT in offspring enhancing effects of the FMTP in sows and four offspring inoculations having a greater impact on some parameters than one, although these effects were not as obvious as perhaps expected. Overall, although the findings of this study demonstrate the impact of early-life intestinal microbiota on host phenotype, FMT, delivered either maternally and/or to offspring (at least the regime used in this study), proved ineffective as a strategy to improve FE in pigs. This was most likely due to adverse effects on the microbiota composition, possibly resulting from the use of nonideal donors. However, possible effects of the antibiotic and/or purgative used as part of the FMT regime in sows cannot be ruled out, and additional work is needed to disentangle the influence of the different components used.

## MATERIALS AND METHODS

### Ethical approval.

The pig study was approved by the Teagasc and Waterford Institute of Technology animal ethics committees and performed according to European Union council directives 91/630/EEC (69) and 98/58/EC (70). An experimental license (AE1932/P032) was obtained from the Irish Health Products Regulatory Authority (HPRA). Piglets that died or were removed during the study were as follows; between birth and weaning, a total of 18 piglets died, 14 due to crushing (CON/CON, *n* = 2; CON/FMT1, *n* = 4; CON/FMT4, *n* = 1; FMT/CON, *n* = 4; FMT/FMT1, *n* = 1; and FMT/FMT4, *n* = 2) and 4 of unknown causes, i.e., normal organs on autopsy (CON/CON, *n* = 1; CON/FMT1, *n* = 1; CON/FMT4, *n* = 1; and FMT/FMT4, *n* = 1). Thirteen piglets were euthanized, 10 due to poor growth (CON/CON, *n* = 2; CON/FMT1, *n* = 1; CON/FMT4, *n* = 2; FMT/CON, *n* = 1; FMT/FMT1, *n* = 2; and FMT/FMT4, *n* = 2), 1 CON/FMT1 due to hemorrhagic lesions on stomach (possibly due to sow crushing), hematoma on right kidney, and mild pneumonia symptoms, and 1 CON/FMT1 and 1 FMT/FMT1 due to broken legs (caused by sow crushing). One pig on the CON/CON treatment died during the study (*post mortem* showed hemorrhage in intestine and spleen), and nine pigs from the following treatments were removed from the study for the following reasons: CON/FMT1, *n* = 1, poor growth; CON/FMT4, *n* = 1, lameness; FMT/CON, *n* = 3, lameness, navel rupture, and pneumonia; FMT/FMT1, *n* = 2, navel rupture; and FMT/FMT4, *n* = 2, navel rupture.

### Fecal microbiota transplantation procedure in sows and sampling.

A schematic showing the preparation of the FMT inoculum from the feces of highly feed-efficient donor pigs is shown in Fig. 1B, and an overview of the sow and offspring treatments and sample collection in Fig. 1A and C. The fecal inoculum was prepared based on a method developed by O’Donnell et al. (71). Fecal samples from 4 finisher pigs (130 days of age) with the lowest RFI (the most feed efficient) from a previous study of 409 pigs (U. M. McCormack et al., submitted) were collected by rectal stimulation directly into sterile bags. Subsamples were collected from each fecal sample, snap-frozen, and stored at −80°C for microbiota analysis. The fecal samples were then placed into anaerobic jars and processed immediately as outlined in Fig. 1B in an anaerobic workstation. Aliquots of 100 ml of the resultant fecal extracts were taken for sow FMT, and aliquots of 8 ml (in 10-ml syringes) were taken for offspring FMT. Subsamples were collected at the start and at the end of the aliquoting process, snap-frozen, and stored at −80°C for microbiota analysis.

At day 60 of gestation, 18 multiparous F1 sows (Large White × Landrace; Hermitage Genetics, Kilkenny, Ireland) were artificially inseminated at random using semen from one of two boars (Hylean Maxgro; Hermitage Genetics) that were selected as having the lowest FCE values (i.e., the most feed efficient). Sows were blocked by boar and body weight and assigned to one of two treatments, (i) control (CON, *n* = 9) and (ii) FMT procedure (FMTP, *n* = 9) (Fig. 1C). “FMTP” refers to all steps used in the procedure, i.e., antibiotic treatment, purgative, fasting, proton-pump inhibitor, and both FMTs, the details of which are outlined below.

On day 61, sows on the FMTP treatment were individually housed, and in order to remove the resident microbiota present, they commenced a 7-day course of a cocktail of antibiotics, administered as top dressing on feed (three antibiotics, used for their broad spectrum of activity: 20 mg/kg/day amoxicillin trihydrate [amoxinsol; Vetoquinol UK Ltd., Buckingham, United Kingdom], 10 mg/kg/day lincomycin-spectinomycin [Linco-Spectin 100; Pfizer, Inc., Cork, Ireland] and 100,000 IU/kg/day colistin [Coliscour; Ceva Sante Animale, Libourne, France]) (Fig. 1A). To ensure that the fecal inoculum was not inactivated by the antibiotics, 3 days were allowed to elapse between the last dose of antibiotic and the first FMT.

On day 68, in order to clear the digestive tract of any feed residue/digesta, FMTP sows received a purgative in two doses (one in the morning and one in the evening) via gastric intubation, which consisted of 145 g Picolax powder (Ferring Ireland Ltd., Dublin, Ireland) dissolved in 1,350 ml water, providing 10 mg sodium picosulfate, 3.5 mg magnesium oxide, and 12 g citric acid per dose. To empty the gastrointestinal tract, sows were fasted for 36 h from the first dose of the purgative until day 70 of gestation, when they received a proton pump inhibitor 1 h prior to FMT (7 tablets per sow, each tablet containing 40 mg omeprazole for a total of 280 mg/sow; Rowex Ltd., Cork, Ireland), to reduce the likelihood of inactivation of the inoculum by gastric acid. Aliquots (100 ml) of the FMT inoculum containing a mean count of 1.4 × 10^9^ CFU/ml, as determined by plating on Wilkins-Chalgren (WC) anaerobe agar (Oxoid Ltd., Basingstoke, Hampshire, United Kingdom), were thawed to room temperature and administered via gastric intubation to each sow in the FMTP group (200 ml/sow, which delivered a dose of 2.8 × 10^11^ CFU or 11.44 log_10_ 16S rRNA gene copies/ng DNA; referred to as the “first FMT”). The gastric tube was then rinsed with a solution of the proton pump inhibitor dissolved in 400 ml lukewarm water. On day 100, FMTP sows received the “second FMT” (containing a mean count of 1.2. × 10^9^ CFU/ml, as determined by plating on WC anaerobe agar, which delivered a dose of 2.5 × 10^11^ CFU or 11.39 log_10_ 16S rRNA gene copies/ng DNA), using the same procedure as on day 70. Fecal samples for microbiota analysis were collected throughout gestation from 6 sows/treatment, as outlined in Fig. 1A, snap-frozen in liquid nitrogen, and stored at −80°C. At the onset of farrowing, the two teats immediately distal to the sow’s head were cleaned with iodine and colostrum was collected manually (from 6 sows/treatment) into sterile containers and stored at −80°C for microbiota analysis.

### Fecal microbiota transplantation, sampling, and management of offspring.

At farrowing, nine piglets (*n* = 162) were selected as having average birth weights for their respective litter, blocked by gender and weight, and randomly assigned to one of three treatments: (i) Control (CON), (ii) FMT at birth (FMT1), and (iii) FMT at birth and days 3, 7, and 28 of age (FMT4) (Fig. 1C). FMT in piglets was performed by orally administering (via syringe) 8 ml of inoculum containing a mean count of 1.2 × 10^9^ CFU/ml (as determined on WC anaerobe agar), which delivered a dose of 9.6 × 10^9^ CFU or 9.97 log_10_ 16S rRNA gene copies/ng DNA.

At weaning, six pigs per litter (2 offspring/treatment; 1 male and 1 female where possible) were selected from 7 litters per sow treatment (*n* = 42 pigs/sow treatment, and *n* = 28 pigs/offspring treatment) (Fig. 1C). All pigs were individually housed from weaning to slaughter (from weaning to 70 days of age in weaner pens [1.2 m by 0.9 m] with plastic slats [Faroex, Manitoba, Canada] and solid plastic dividers between pens, and from 70 to 155 days of age in fully slatted finisher pens [1.81 m by 1.18 m] with solid plastic panel partitions) and fed a common sequence of the same diets (see Table S4 in the supplemental material). Body weight and feed disappearance were recorded weekly to calculate performance (ADFI, ADG, FCE, and RFI).

10.1128/mSystems.00134-17.9 TABLE S4  Compositions of all diets used in the study (g/kg). Download TABLE S4, DOCX file, 0.02 MB. Copyright © 2018 McCormack et al. 2018 McCormack et al. This content is distributed under the terms of the Creative Commons Attribution 4.0 International license.

Throughout the study, fecal samples were collected from 36 pigs (1 pig/treatment/litter [same gender per litter] from six litters per sow treatment) by rectal stimulation at four time points for microbiota analysis (Fig. 1A and C). Intact litters were selected to control for interlitter and intersow variation.

At ~155 days of age, all pigs were slaughtered by CO_2_ stunning followed by exsanguination. Hot carcass weight was recorded immediately, and cold carcass weight and kill out percentage calculated (4). Back fat and muscle depth were recorded and used to estimate lean meat yield (4). For microbiota and VFA analyses, ileal and cecal digesta samples were collected as described previously (4) from the 36 selected pigs, and colon digesta was sampled 1 m distal to the cecum. Intestinal tissue was sampled from the duodenum, jejunum, and ileum for histological analysis (72). Using a glass slide, mucosal scrapings were collected from 10 cm of duodenum tissue (5 cm distal to the location of the histological sample) for gene expression and brush border enzyme activity analyses. All samples, except those for histological analysis, were snap-frozen in liquid nitrogen and stored at −80°C until processing.

Blood for hematology and biochemistry analyses was collected from the 36 selected pigs during exsanguination (Fig. 1C) using Vacuette tubes (Labstock, Dublin, Ireland) as described previously (7).

### Microbiota analysis in inocula and fecal, digesta, and colostrum samples.

Total DNA was extracted from all samples collected throughout the study from the inocula (donors and aliquoted and thawed inocula), sows, and offspring, using the QIAamp DNA stool minikit (Qiagen, Crawley, United Kingdom) according to the manufacturer’s instructions, apart from adding a bead-beating step and increasing the lysis temperature to 95°C (73). Total DNA was extracted from colostrum using the PowerFood microbial DNA isolation kit (Cambio, Cambridge, England) according to the manufacturer’s instructions.

The V3–V4 region of the 16S rRNA gene (~460 bp) was sequenced (2 × 250 bp) using the Illumina MiSeq, following a standard protocol as previously described (4). Sequence reads were checked for quality using FastQC software, and adapters were removed (Illumina CLIP software). Reads were then trimmed to 240 bp at the end of the sequence using Trimommatic version 0.36 (74), forward and reverse reads were merged using Flash, and quality checks performed to guarantee maximum coverage. Reads were then clustered into operational taxonomical units (OTUs) using a 97% sequence identity threshold, and chimeras removed with the CD-HIT-OTU pipeline. The Ribosomal Database Project classifier (RDP) was used (75) for taxonomic assignment, with a cutoff of 80%, with those taxa <80% labeled as unclassified. Samples with <5,000 total joined reads were excluded from the analyses, except for colostrum samples, for which a cutoff of >1,000 reads was applied. The OTU data were scaled to the minimum number of total reads for each sample type and filtered to remove OTUs at <100 reads. Alpha diversity (Chao1 [OTU richness] and Shannon and Simpson [OTU richness and abundance] diversities) and beta dispersion estimates were calculated using the Adonis2 and beta permutation functions in the Vegan package in R, each with 999 permutations. Principal component analysis (PCA) plots were generated with the bioconductor package DESeq2 and ggplot in R. Heatmaps of relative abundance were generated in GraphPad Prism 7.

Quantification of the 16S rRNA gene was performed in triplicate for all inoculum, fecal, and digesta samples by quantitative PCR (qPCR) in order to estimate the total bacterial load, as described previously (4).

### Prediction of microbial function.

The functionality of the microbiota for each sample, based on 16S rRNA data and the 13_5 version of the Greengenes database for taxonomy and OTU assignments, was predicted *in silico* using PICRUSt. Prediction of functions was inferred based on Kyoto Encyclopedia of Genes and Genomes (KEGG) annotations. Pathways not related to bacteria, not relevant to porcine studies, and for which the relative abundance in samples was <0.001% were dismissed.

### Volatile fatty acid analysis and pH of digesta samples.

Volatile fatty acid concentrations were measured in ileal, cecal, and colonic digesta samples in triplicate using gas chromatography, as described previously (4). Briefly, ~8 g of sample was weighed, the pH recorded, and a trichloroacetic acid (TCA) extraction performed. Extracts were mixed with an internal standard, and 1-µl volumes injected into the gas chromatograph (Agilent 5890) under the following conditions: hydrogen at 30 lb/in^2^, helium at 50 lb/in^2^, and temperatures of 80°C for the oven, 280°C for the detector, and 250°C for the injector.

### Intestinal histology.

Tissue samples (~3-cm sections) collected from the duodenum, jejunum, and ileum at slaughter were processed for histological analysis as described previously (4). Ten villi were examined per slide for villus height and width, crypt depth, and goblet cell number using a light microscope at ×400 magnification.

### Candidate gene expression and brush border enzyme activity in the duodenum.

Total RNA was isolated from 20 mg duodenal mucosal scrapings using mechanical homogenization and the RNeasy minikit (Qiagen, Hilden, Germany). Samples were homogenized using the FastPrep-24 instrument (MP Biomedicals, Santa Ana, CA, USA). Genomic DNA was removed, and the RNA was quantified and evaluated and cDNA synthesized as previously outlined (76). The candidate genes measured by qPCR and the primers used are listed in Table S5. Amplifications were performed in 20-µl reaction mixtures on a real-time PCR Mx3000P thermocycler (Agilent Technologies, Waghaeusel-Wiesental, Germany) in duplicate, as described previously (76).

10.1128/mSystems.00134-17.10 TABLE S5  Forward and reverse primers used for quantitative PCR, PCR efficiencies, and correlation coefficients of standard curves. Download TABLE S5, DOCX file, 0.02 MB. Copyright © 2018 McCormack et al. 2018 McCormack et al. This content is distributed under the terms of the Creative Commons Attribution 4.0 International license.

Duodenal maltase (EC 3.2.1.20), saccharase (EC 3.2.1.48), and lactase (EC 3.2.1.23) activities (expressed as micromoles of substrate hydrolyzed per minute per gram of protein [U/g protein]) were analyzed as described previously (77).

### Hematology and blood biochemistry analyses.

Hematological analysis was performed using a Beckman Coulter Ac-T diff analyzer (Beckman Coulter, High Wycombe, United Kingdom). Total protein, blood urea nitrogen, cholesterol, glucose, triglycerides, creatinine, and creatine kinase concentrations were determined in serum samples using an ABX Pentra 400 clinical chemistry analyzer (Horiba, Northampton, United Kingdom) calibrated according to the manufacturer’s instructions, with every fifth sample run in duplicate to determine analyzer accuracy.

### Statistical analysis.

All data were statistically analyzed using SAS 9.3, using gender, treatment, boar, and time point, where appropriate (weekly measures of growth performance), as fixed effects. Pig nested within sow was used as a random effect. The qPCR data were log_10_ transformed prior to statistical analysis, which was performed using a generalized linear mixed model (PROC GLIMMIX).

Microbial composition and predicted functionality data were analyzed using generalized linear mixed model equation methods in PROC GLIMMIX. A gamma distribution was assumed for all data. Models for sow feces and colostrum included sow treatment, fecal sample time point (if applicable), and their interactions as fixed effects. Offspring models included sow treatment, offspring treatment, fecal sample time point, and their interactions as fixed effects. Additionally, a random intercept for each time point was included (repeated measure). A similar model was used for digesta samples collected at slaughter, but intestinal site was included instead of fecal sample time point. In all models, data were back transformed to the original distribution using the *ilink* option. Spearman rank-order correlations were performed between relative abundances of bacterial taxa at the genus level for each sample type and according to sow/offspring treatment, with body weight at slaughter, using the PROC CORR procedure, using the step-down Bonferroni test to correct for multiple comparisons. Heatmaps were produced in GraphPad Prism 7. Principal components were calculated from regularized log-transformed counts and plotted using ggplot2, and the DESeq2 package was used to calculate differential abundance by use of negative binomial generalized linear models. The alpha levels for determination of significance and trends were 0.05 and 0.10, respectively.

### Accession number(s).

The raw 16S rRNA gene sequence data generated from this study are available in the European Nucleotide Archive under accession number PRJEB22181.
