# Supplementary material for: Fecal Microbiota Transplantation in Gestating Sows and Neonatal Offspring Alters Lifetime Intestinal Microbiota and Growth in Offspring
Source: mSystems. 2018 Mar 13;3(3):e00134-17. doi: 10.1128/mSystems.00134-17 (PMC5864416; doi:10.1128/mSystems.00134-17)
Supplement: TABLE S1 [file sys001182193st1.docx]

| **Sow treatment** | **Control^2^** | | |  | **FMTP^3^** | | |  | **S.E.M** | **P-value** | | |
| --- | --- | --- | --- | --- | --- | --- | --- | --- | --- | --- | --- | --- |
| **Offspring treatment** | **Control^4^** | **FMT1^5^** | **FMT4^6^** |  | **Control** | **FMT1** | **FMT4** |  |  | **Interaction** | **Sow** | **Offspring** |
| **Body weight (Kg)** | | | | | | | | | | | | |
| Day 70 | 35.2^a^ | 29.9^b^ | 30.4^b^ |  | 29.1^b,c^ | 27.1^c,d^ | 26.4^d^ |  | 2.29 | 0.01 | 0.02 | 0.19 |
| Day 155 | 127.9^a^ | 116.0^c^ | 120.9^b^ |  | 115.3^c^ | 114.2^c^ | 111.3^c^ |  | 2.87 | <0.001 | <0.001 | <0.001 |
| **Residual Feed Intake (g/day)** | | | | | | | | | | | | |
| Weaning to Day 155 | 21.2^A^ | -30.2^B^ | 24.6^A^ |  | -30.1^B^ | 9.4^A,B^ | 1.33^A,B^ |  | 21.38 | 0.08 | 0.51 | 0.52 |
| **Alpha diversity (Shannon index)** | | | | | | | | | | | | |
| Faeces day 50 | 3.49^b^ | 3.21^b^ | 3.73^a,b^ |  | 3.70^a,b^ | 4.13^a^ | 4.22^a^ |  | 0.226 | 0.02 | 0.004 | 0.22 |
| Ileum | 1.80^b^ | 2.74^a^ | 1.84^b^ |  | 1.71 ^b^ | 1.83^b^ | 1.71^b^ |  | 0.123 | <0.001 | <0.001 | <0.001 |
| **Bacterial load (log_10_ copies of 16S rRNA gene/ng DNA)** | | | | | | | | | | | | |
| Ileum | 4.35^a,b^ | 3.97^a,b^ | 3.91^b^ |  | 4.53^a^ | 3.99^a,b^ | 4.04^a,b^ |  | 0.115 | <0.001 | 0.28 | <0.001 |
| **Microbiota composition [median relative abundance (%)]** | | | | | | | | | | | | |
| **Faeces at weaning** | | | | | | | | | | | | |
| G_*Asteroleplasma* | 0.51^b^ | 0.57^b^ | 0.17^b^ |  | 2.09^a^ | 1.76^a,b^ | 1.89^a,b^ |  | 0.06 | <0.001 | <0.001 | 0.34 |
| G_*Blautia* | 1.46^a,b^ | 1.00^a,b^ | 0.45^b^ |  | 0.59^a,b^ | 1.67^a,b^ | 3.32^a^ |  | 0.22 | 0.008 | 0.06 | 0.59 |
| G_*Faecalibacterium* | 0.54^c^ | 1.09^a^ | 0.74^a^ |  | 0.38^b^ | 0.19^b^ | 0.82^a^ |  | 0.10 | 0.05 | 0.02 | 0.29 |
| G_*Oribacterium* | 1.58^a^ | 1.39^a^ | 5.04^a^ |  | 0.38^b^ | 0.14^b^ | 1.04^a^ |  | 0.07 | <0.001 | <0.001 | 0.14 |
| P_*Planctomycetes* | 2.72^a^ | 0.37^b^ | 1.43^a,b^ |  | 1.52^a,b^ | 0.39^b^ | 0.83^a,b^ |  | 0.16 | 0.009 | 0.29 | 0.001 |
| **Faeces at day 50** | | | | | | | | | | | | |
| P_*Actinobacteria* | 0.10^a,b^ | 0.05^b^ | 0.11^a,b^ |  | 0.13^a,b^ | 0.27^a,b^ | 1.08^a^ |  | 0.015 | <0.001 | <0.001 | 0.04 |
| G_*Anaerostipes* | 0.021^a,b^ | 0.002^b^ | 0.189^a,b^ |  | 0.019^a,b^ | 0.056^a,b^ | 0.451^a^ |  | 0.004 | 0.003 | 0.06 | 0.002 |
| G_*Asteroleplasma* | 0.90^a,b^ | 0.33^a,b^ | 0.64^a,b^ |  | 0.07^b^ | 0.45^a,b^ | 1.05^a^ |  | 0.026 | 0.002 | 0.14 | 0.06 |
| G_*Campylobacter* | 0.76^a,b^ | 0.22^b^ | 0.54^a,b^ |  | 0.96^a,b^ | 1.44^a^ | 1.43^a^ |  | 0.104 | 0.003 | 0.004 | 0.37 |
| G_*Alistipes* | 0.27^b^ | 0.14^b^ | 0.18^b^ |  | 0.33^a,b^ | 0.59^a^ | 0.70^a^ |  | 0.066 | 0.01 | <0.001 | 0.85 |
| G_*Peptococcus* | 0.22^b^ | 0.08^b^ | 0.12^b^ |  | 0.12^b^ | 0.14^b^ | 2.13^a^ |  | 0.033 | <0.001 | 0.79 | 0.003 |
| P_*Tenericutes* | 0.90^a,b^ | 0.33^a,b^ | 0.64^a,b^ |  | 0.07^b^ | 0.46^a,b^ | 1.04^a^ |  | 0.025 | 0.001 | 0.89 | 0.59 |
| **Faeces at day 65** | | | | | | | | | | | | |
| G_*Alistipes* | 0.32^a,b^ | 0.16^b^ | 0.47^a^ |  | 0.68^a^ | 0.55^a^ | 0.14^b^ |  | 0.072 | 0.005 | 0.34 | 0.22 |
| G_*Anaerostipes* | 0.51^s^ | 0.16^s,b^ | 0.65^a^ |  | 0.02^b^ | 0.84^a^ | 0.24^a^ |  | 0.005 | <0.001 | 0.07 | 0.06 |
| G_*Campylobacter* | 0.37^b^ | 0.39^b^ | 0.98^a,b^ |  | 1.62^a^ | 1.47^a^ | 0.79^a,b^ |  | 0.182 | 0.002 | 0.01 | 0.88 |
| G_*Terrisporobacter* | 0.13^a^ | 0.03^a^ | 0.12^a^ |  | 0.07^a^ | 0.01^b^ | 0.01^b^ |  | 0.001 | <0.001 | 0.008 | 0.008 |
| G_*Peptococcus* | 0.22^b^ | 0.08^b^ | 0.12^b^ |  | 0.12^b^ | 0.14^b^ | 2.13^a^ |  | 0.033 | 0.001 | 0.64 | 0.001 |
| G_*Ruminococcus* | 0.30^a,b^ | 0.31^a,b^ | 0.24^a,b^ |  | 1.11^a^ | 0.47^a,b^ | 0.13^b^ |  | 0.067 | 0.001 | 0.17 | 0.003 |
| **Faeces at day 100** | | | | | | | | | | | | |
| G_*Peptococcus* | 0.13^a,b^ | 0.06^b^ | 0.25^a^ |  | 0.27^a^ | 0.15^a,b^ | 0.84^a^ |  | 0.057 | <0.001 | 0.65 | <0.001 |
| **Ileum** | | | | | | | | | | | | |
| P_*Actinobacteria* | 0.07^b^ | 0.64^a,b^ | 0.85^a^ |  | 0.10^a,b^ | 0.17^a,b^ | 0.04^b^ |  | 0.015 | <0.001 | 0.001 | 0.53 |
| G_*Blautia* | 0.09^b^ | 1.97^a^ | 0.09^b^ |  | 0.01^c^ | 0.11^b^ | 0.12^b^ |  | 0.005 | <0.001 | <0.001 | <0.001 |
| G_*Butyricicoccus* | 0.064^a^ | 0.368^a^ | 0.062^a^ |  | 0.005^b^ | 0.234^a^ | 0.096^a^ |  | 0.0023 | <0.001 | 0.55 | <0.001 |
| G_*Butyricimonas* | 0.05^b^ | 0.89^a^ | 0.03^b^ |  | 0.04^b^ | 0.09^b^ | 0.05^b^ |  | 0.018 | <0.001 | 0.006 | <0.001 |
| P_*Spirochaetaes* | 11.41^a,b^ | 19.41^a,b^ | 7.27^b^ |  | 24.07^a^ | 10.31^a,b^ | 9.40^a,b^ |  | 4.680 | 0.004 | 0.52 | 0.67 |
| P_*Chlamydiae* | 0.18^a,b^ | 4.30^a^ | 0.14^a,b^ |  | 0.04^b^ | 0.38^a,b^ | 0.22^a,b^ |  | 0.020 | <0.001 | <0.001 | <0.001 |
| G_*Chlamydia* | 0.18^b,c^ | 4.29^a^ | 0.14^b,c^ |  | 0.04^c^ | 0.38^b^ | 0.22^b,c^ |  | 0.021 | <0.001 | 0.17 | 0.35 |
| G_*Faecalibacterium* | 0.04^a,b^ | 0.21^a^ | 0.03^a,b^ |  | 0.01^b^ | 0.09^a,b^ | 0.05^a,b^ |  | 0.005 | 0.002 | 0.14 | 0.002 |
| G_*Oscillibacter* | 0.05^c^ | 0.07^c^ | 0.23^b^ |  | 1.63^a^ | 0.79^a^ | 0.18^b^ |  | 0.022 | <0.001 | <0.001 | 0.70 |
| G_*Prevotella* | 8.62^a,b^ | 4.31^b^ | 5.82^b^ |  | 10.93^a,b^ | 9.12^a,b^ | 19.02^a^ |  | 2.281 | 0.03 | 0.005 | 0.20 |
| G_*Sphaerochaeta* | 5.52^b^ | 8.99^a,b^ | 1.80^b^ |  | 17.19^a^ | 3.76^b^ | 5.71^b^ |  | 0.821 | 0.01 | 0.17 | 0.04 |
| **Predicted microbial function in the ileum [median relative abundance (%)]** | | | | | | | | | | | | |
| Alpha-linolenic acid | 0.51^b^ | 5.79^a^ | 5.41^a^ |  | 5.61^a^ | 0.91^b^ | 0.63^b^ |  | 0.174 | <0.001 | 0.03 | 0.56 |
| Tryptophan metabolism | 3.02^a,b^ | 3.87^a^ | 2.45^b^ |  | 3.86^a,b^ | 2.97^a,b^ | 2.74^a,b^ |  | 2.072 | 0.002 | 0.67 | 0.004 |
| Protein kinases | 3.06^a,b^ | 3.76^a^ | 2.57^b^ |  | 3.44^a,b^ | 3.17^a,b^ | 2.83^a,b^ |  | 2.299 | <0.001 | 0.79 | <0.001 |
| Pentose and glucuronate interconversions | 3.09^a,b^ | 3.19^a,b^ | 2.28^b^ |  | 4.05^a^ | 3.09^a,b^ | 3.22^a,b^ |  | 1.936 | <0.001 | 0.008 | 0.88 |
| Propanoate metabolism | 3.19^a,b^ | 3.14^a,b^ | 2.62^b^ |  | 3.58^a^ | 3.31^a,b^ | 2.93^a,b^ |  | 0.387 | <0.001 | 0.57 | 0.001 |
| Primary and secondary bile acid biosynthesis | 2.93^b^ | 7.30^a^ | 2.97^b^ |  | 1.48^b^ | 2.84^b^ | 1.29^b^ |  | 0.842 | <0.001 | <0.001 | <0.001 |
| Steroid biosynthesis | 1.23^b^ | 6.43^a^ | 7.14^a^ |  | 3.89^a,b^ | 2.34^a,b^ | 1.27^b^ |  | 0.579 | <0.001 | <0.001 | <0.001 |
| Pentose phosphate pathway | 3.11^a,b^ | 3.35^a^ | 2.66^b^ |  | 3.60^a^ | 3.12^a,b^ | 2.98^s,b^ |  | 0.448 | <0.001 | 0.08 | 0.001 |
| Pyruvate metabolism | 3.07^a,b^ | 3.46^a^ | 2.49^b^ |  | 3.71^a^ | 3.06^a,b^ | 3.07^a,b^ |  | 2.224 | 0.001 | 0.07 | 0.005 |
| Tetracycline biosynthesis | 2.80^a,b^ | 4.21^a^ | 1.86^b^ |  | 4.34^a^ | 2.96^a,b^ | 2.85^a,b^ |  | 1.490 | <0.001 | 0.08 | <0.001 |
| Styrene degradation | 1.46^b^ | 6.22^a^ | 1.82^b^ |  | 2.07^a,b^ | 2.40^a,b^ | 5.16^a^ |  | 1.376 | <0.001 | 0.38 | <0.001 |
| Various types of N-glycan biosynthesis | 1.32^c^ | 0.66^c^ | 22.34^a^ |  | 15.48^a^ | 5.01^b^ | 1.33^c^ |  | 0.306 | <0.001 | 0.06 | 0.06 |
| Stilbenoid, diarylheptanoid and gingerol biosynthesis | 2.10^b^ | 1.57^b^ | 2.36^b^ |  | 38.42^a^ | 1.57^b^ | 1.57^b^ |  | 0.684 | <0.001 | 0.64 | <0.001 |
| **Volatile fatty acids (µmol/g digesta)** | | | | | | | | | | | | |
| **Ileum** |  |  |  |  |  |  |  |  |  |  |  |  |
| Propionic acid | 26.8^a,b^ | 19.9^b^ | 14.9^b^ |  | 18.9^b^ | 42.7^a^ | 27.7^a,b^ |  | 8.25 | 0.02 | 0.03 | 0.33 |
| Butyric acid | 7.1^b^ | 33.9^a^ | 23.8^a^ |  | 13.5^b^ | 11.9^b^ | 13.7^b^ |  | 4.29 | 0.001 | 0.12 | 0.01 |
| Isovaleric acid | 16.7^a,b^ | 13.1^a,b^ | 24.0^a^ |  | 14.7^a,b^ | 20.2^a^ | 8.3^b^ |  | 5.63 | 0.03 | 0.37 | 0.91 |
| **Intestinal histology** | | | | | | | | | | | | |
| **Duodenum** | | | | | | | | | | | | |
| Goblet cell (number per villi) | 41^a^ | 36^a,b^ | 39^a^ |  | 34^b^ | 34^b^ | 33^b^ |  | 1.8 | 0.05 | 0.004 | 0.63 |
| **Ileum** | | | | | | | | | | | | |
| Villus height (µm) | 545^a,b^ | 602^a^ | 585^a,b^ |  | 452^a,b^ | 413^b^ | 443^a,b^ |  | 33.1 | 0.001 | 0.05 | 0.51 |
| **Gene expression (Fold change^7^)** | | | | | | | | | | | | |
| *MCT1* | 0.00^a,b^ | 0.24^a^ | 0.08^a,b^ |  | 0.08^a,b^ | 0.03^b^ | 0.17^a,b^ |  | - | 0.04 | 0.91 | 0.17 |
| *SMCT* | 0.00^a,b^ | -0.22^b^ | 0.06^a,b^ |  | -0.14^a,b^ | 0.16^a^ | -0.17^a,b^ |  | - | 0.003 | 0.78 | 0.74 |
| **Haematology** | | | | | | | | | | | | |
| Monocyte (no. x 10^3^ cells/µL) | 0.52^b^ | 0.78^a,b^ | 0.77^a,b^ |  | 0.83^a^ | 0.66^a,b^ | 0.68^a,b^ |  | 0.083 | 0.02 | 0.59 | 0.79 |
| Red cell distribution width (fL) | 19.4^a,b^ | 20.4^a^ | 18.5^b^ |  | 19.4^a,b^ | 18.7^b^ | 19.1^a,b^ |  | 0.44 | 0.04 | 0.37 | 0.19 |

^1^Least squares means and pooled standard errors of the mean are presented.

Sows were assigned to one of two treatment groups: ^2^Control (Control; n=9) and ^3^FMT procedure (FMTP; n=9); FMTP sows received FMT via gastric intubation on days 70 and 100 of gestation. Piglets were assigned to one of three treatment groups at birth: ^4^Control; ^5^FMT1 (FMT at birth) and ^6^FMT4 (FMT at birth and days 3, 7 and 28 of age). Data from 36 pigs: Sow treatment level control n=18; FMTP n=18; Offspring treatment level control n=12; FMT1 n=12; FMT4 n=12. ^7^Values represent log_10_-fold changes relative to Control sow × Control offspring after normalization to glyceraldehyde 3-phosphate dehydrogenase (*GAPDH*), Beta-actin (*ACTB*) and Beta-2 microglobulin (*B2M*) gene expression.

^a, b, c^ Within each row, values that do not share a common superscript are significantly different due to a sow × offspring treatment interaction (P≤0.05).

^A, B, C^ Within each row, values that do not share a common superscript tended to be significantly different due to a sow × offspring treatment interaction (0.05≤P≤0.10).
