# Supplementary figures and images for: Fecal Microbiota Transplantation in Gestating Sows and Neonatal Offspring Alters Lifetime Intestinal Microbiota and Growth in Offspring
Source: mSystems. 2018 Mar 13;3(3):e00134-17. doi: 10.1128/mSystems.00134-17 (PMC5864416; doi:10.1128/mSystems.00134-17)

## A. Phylum level

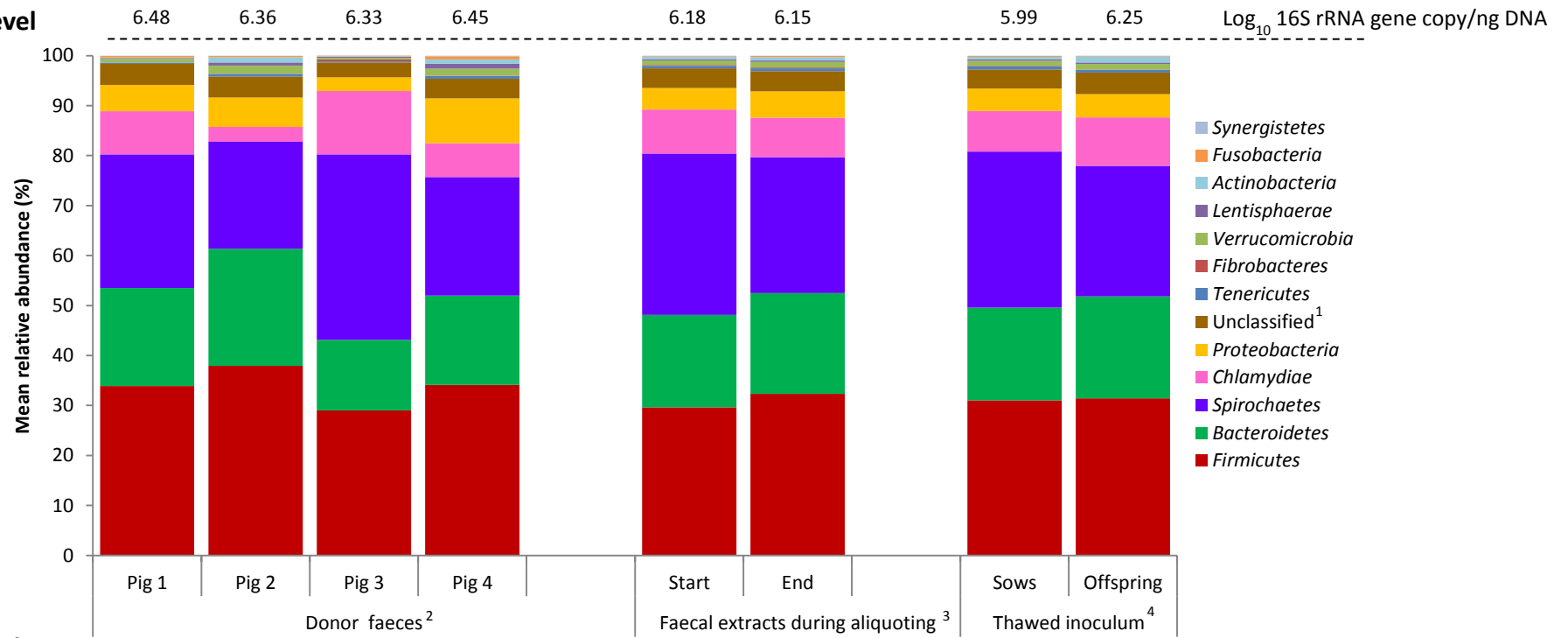

## B. Genus level

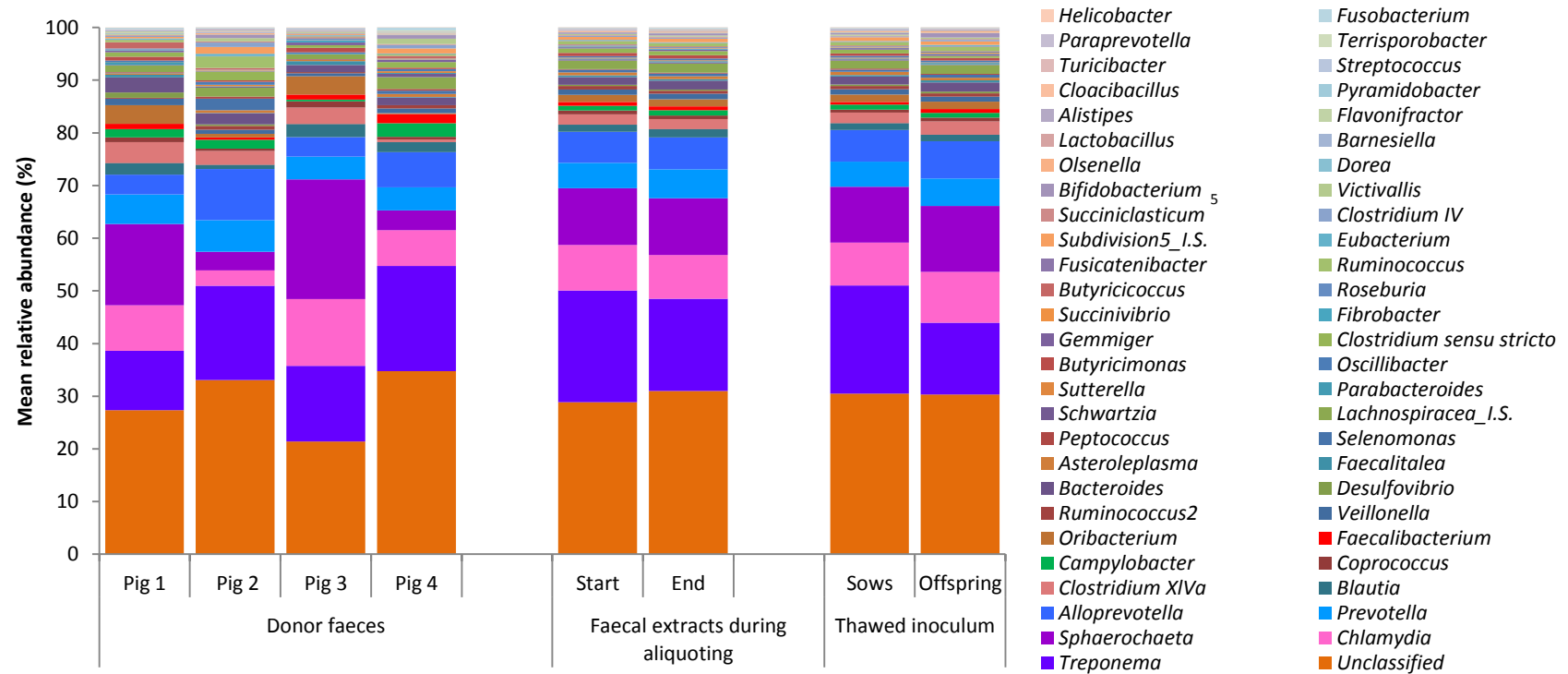

Supplement: FIG S1 [file sys001182193sf1.pdf]

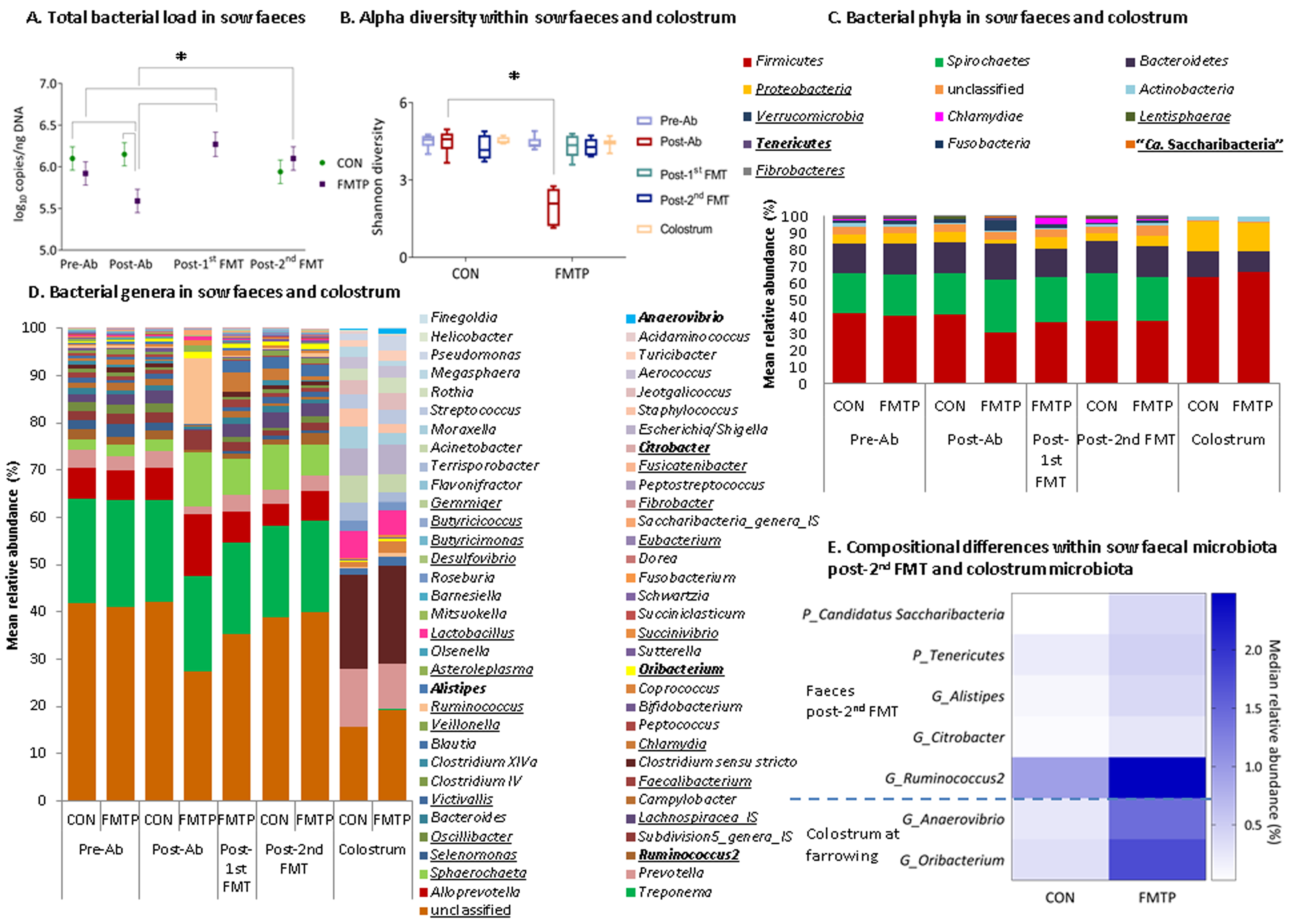

Supplement: FIG S2 [file sys001182193sf2.tif]

**A. Bacterial load in offspring faeces**

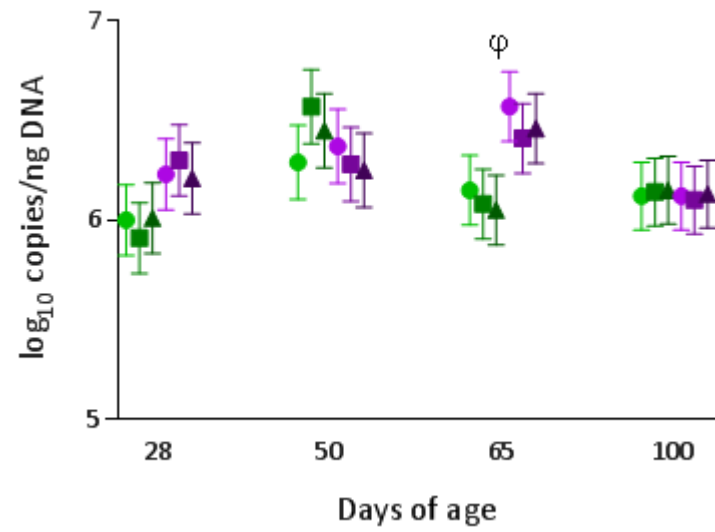

**B. Bacterial load in offspring digesta**

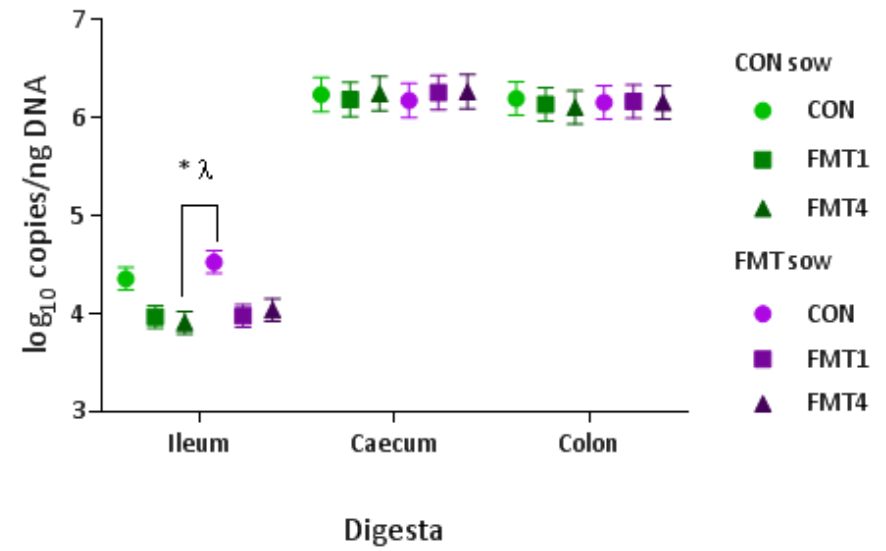

Supplement: FIG S4 [file sys001182193sf4.pdf]

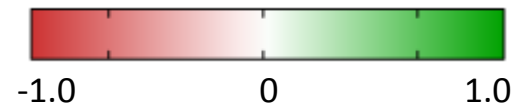

Spearman correlation coefficient

### A. Sow treatment level

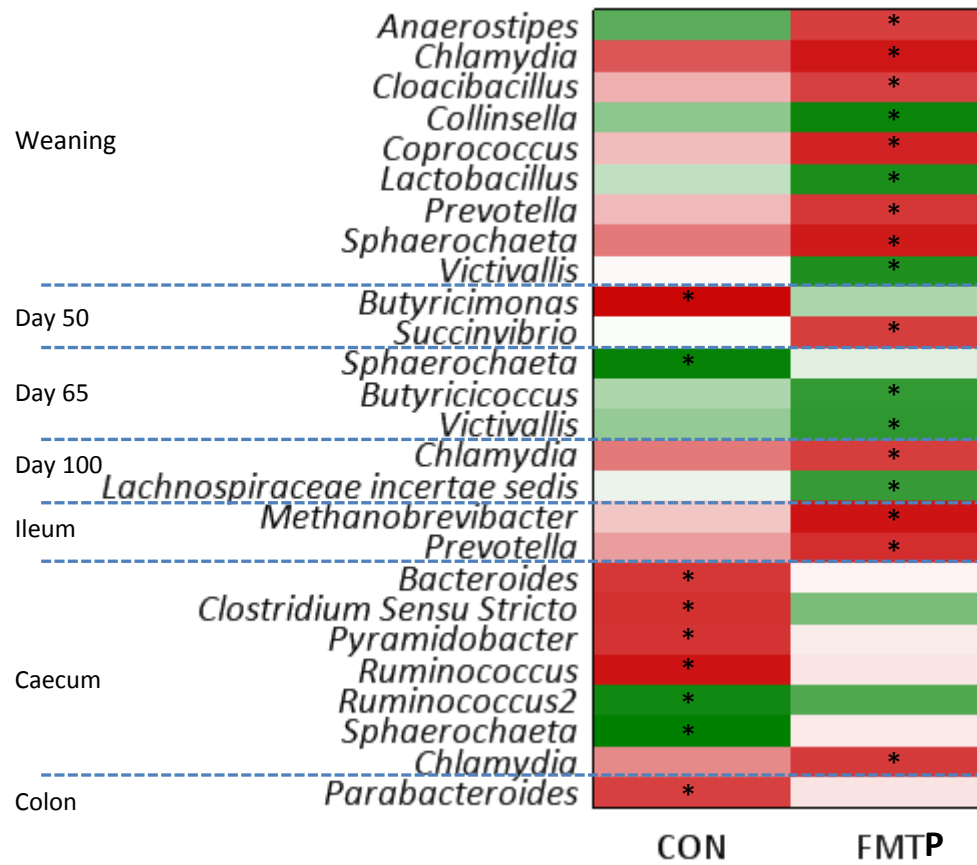

### B. Offspring treatment level

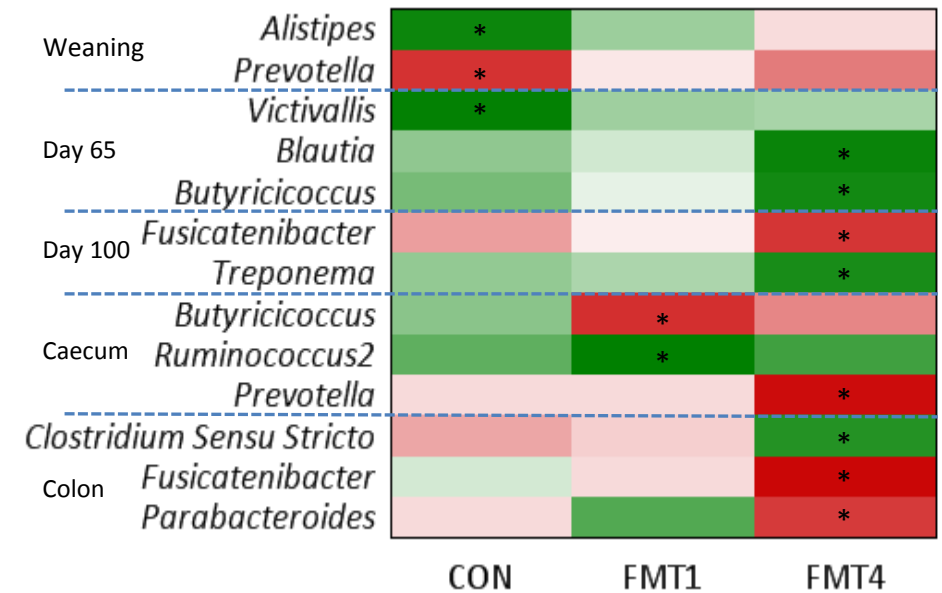

Supplement: FIG S5 [file sys001182193sf5.pdf]
