# Supplementary material for: Fecal Microbiota Transplantation in Gestating Sows and Neonatal Offspring Alters Lifetime Intestinal Microbiota and Growth in Offspring
Source: mSystems. 2018 Mar 13;3(3):e00134-17. doi: 10.1128/mSystems.00134-17 (PMC5864416; doi:10.1128/mSystems.00134-17)
Supplement: TABLE S2 [file sys001182193st2.docx]

| **Genus (Phylum)** | **CON^1^** | **FMTP^2^** | **Confidence interval** | | **P-value** |
| --- | --- | --- | --- | --- | --- |
|  |  |  | **Lower** | **Upper** |  |
| **Pre-antibiotic faeces** |  |  |  |  |  |
| *Butyricimonas* (*Bacteroidetes*) | 0.06 | 0.16 | 0.036 | 0.282 | 0.03 |
| *Fusobacterium* (*Fusobacteria*) | 0.12 | 0.04 | 0.025 | 0.254 | 0.04 |
| *Roseburia* (*Firmicutes*) | 0.22 | 0.02 | 0.002 | 0.848 | 0.02 |
| *Schwartzia* (*Firmicutes*) | 0.95 | 0.19 | 0.048 | 0.395 | 0.02 |
| **Post-antibiotic faeces** |  |  |  |  |  |
| *Asteroleplasma* (*Tenericutes*) | 0.3 | 2.5 | 0.17 | 4.27 | <0.0001 |
| *Butyricicoccus* (*Firmicutes*) | 0.1 | 1.1 | 0.02 | 6.19 | 0.003 |
| *Butyricimonas* (*Bacteroidetes*) | 0.1 | 0.2 | 0.04 | 0.41 | 0.03 |
| *Chlamydia* (*Chlamydiae*) | 0.8 | 0.1 | 0.02 | 4.06 | 0.01 |
| *Citrobacter* (*Proteobacteria*) | 0.03 | 0.29 | 0.016 | 1.069 | 0.004 |
| *Desulfovibrio* (*Proteobacteria*) | 0.1 | 0.9 | 0.06 | 2.23 | 0.0002 |
| *Eubacterium* (*Firmicutes*) | 0.12 | 0.01 | 0.001 | 0.239 | 0.001 |
| *Faecalibacterium* (*Firmicutes*) | 0.82 | 0.01 | 0.003 | 1.326 | <0.0001 |
| *Fibrobacter* (*Fibrobacteres*) | 0.044 | 0.003 | 0.0006 | 0.0917 | 0.002 |
| *Fusicatenibacter* (*Firmicutes*) | 0.03 | 0.01 | 0.001 | 0.069 | 0.02 |
| *Gemmiger* (*Proteobacteria*) | 0.06 | 0.01 | 0.001 | 0.147 | 0.05 |
| *Lachnospiraceae Incertae Sedis* (*Firmicutes*) | 2.7 | 0.5 | 0.25 | 4.15 | <0.0001 |
| *Lactobacillus* (*Firmicutes*) | 0.3 | 2.6 | 0.16 | 5.85 | <0.0001 |
| *Oribacterium* (*Firmicutes*) | 0.5 | 1.7 | 0.24 | 3.55 | 0.01 |
| *Oscillibacter* (*Firmicutes*) | 1.95 | 0.04 | 0.009 | 4.391 | <0.0001 |
| *Ruminococcus* (*Firmicutes*) | 0.5 | 16.7 | 0.2 | 29.39 | <0.0001 |
| *Ruminococcus2* (*Firmicutes*) | 1.8 | 0.4 | 0.21 | 3.43 | 0.003 |
| *Selenomonas* (*Firmicutes*) | 1.58 | 0.01 | 0.002 | 3.111 | <0.0001 |
| *Sphaerochaeta* (*Spirochaetes*) | 2.6 | 11.5 | 1.17 | 25.83 | 0.01 |
| *Succinivibrio* (*Proteobacteria*) | 0.2 | 1.6 | 0.13 | 3.25 | 0.0003 |
| *Unclassified* (*Grouped^3^*) | 42.3 | 27.4 | 22.12 | 52.24 | 0.006 |
| *Veillonella* (*Firmicutes*) | 0.5 | 0.1 | 0.06 | 0.94 | 0.03 |
| *Victivallis* (*Lentisphaerae*) | 0.97 | 0.01 | 0.001 | 1.886 | <0.0001 |
