# Supplementary material for: Fecal Microbiota Transplantation in Gestating Sows and Neonatal Offspring Alters Lifetime Intestinal Microbiota and Growth in Offspring
Source: mSystems. 2018 Mar 13;3(3):e00134-17. doi: 10.1128/mSystems.00134-17 (PMC5864416; doi:10.1128/mSystems.00134-17)
Supplement: FIG S3 [file sys001182193sf3.pdf]

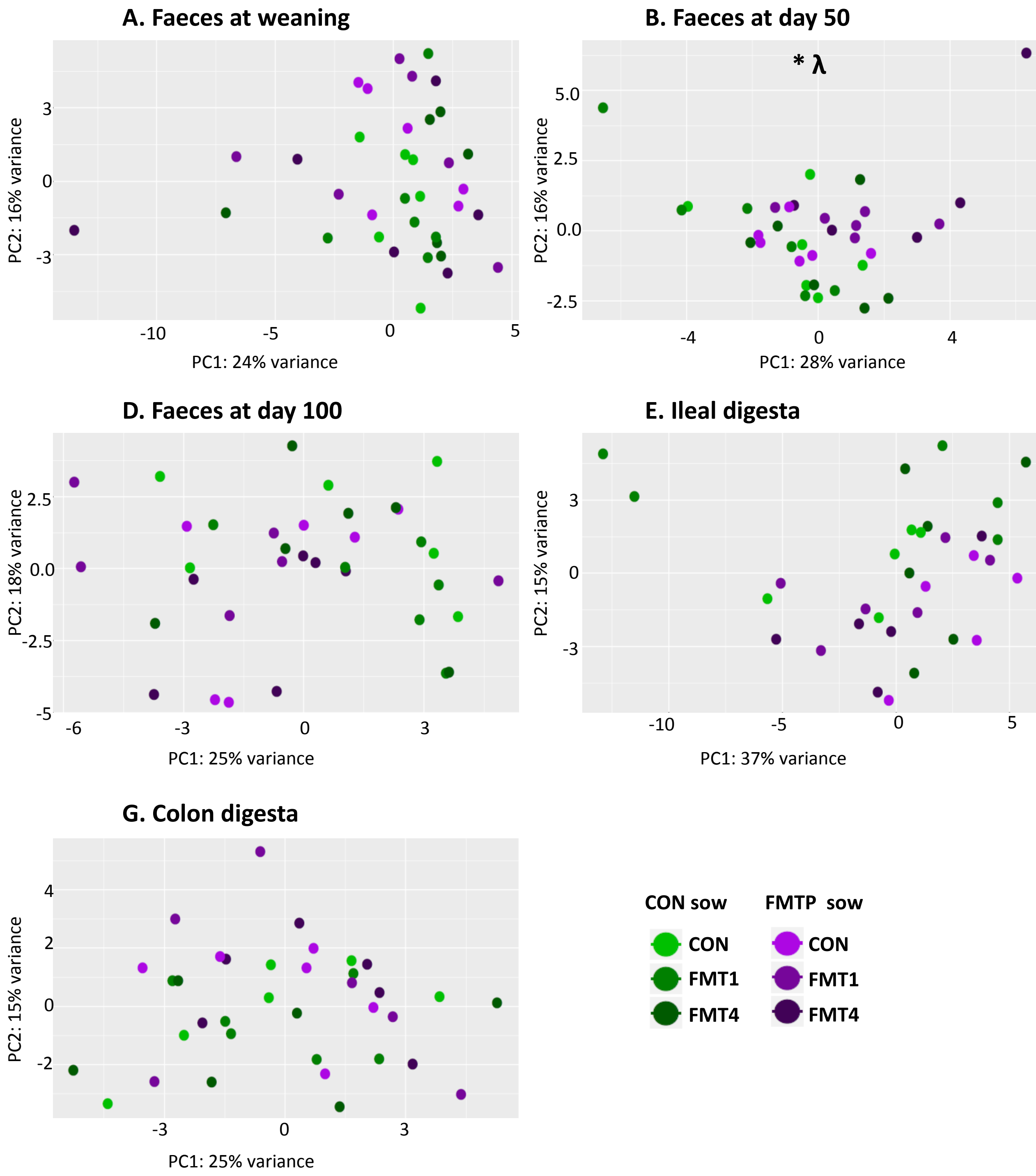

A. Faeces at weaning
PC2: 16% variance
PC1: 24% variance
B. Faeces at day 50
* λ
PC2: 16% variance
PC1: 28% variance
C. Faeces at day 65
PC2: 19% variance
PC1: 26% variance
D. Faeces at day 100
PC2: 18% variance
PC1: 25% variance
E. Ileal digesta
PC2: 15% variance
PC1: 37% variance
F. Caecal digesta
* φ
PC2: 16% variance
PC1: 25% variance
G. Colon digesta
PC2: 15% variance
PC1: 25% variance
CON sow
CON
FMT1
FMT4
FMTP sow
CON
FMT1
FMT4
