# Supplementary material for: Fecal Microbiota Transplantation in Gestating Sows and Neonatal Offspring Alters Lifetime Intestinal Microbiota and Growth in Offspring
Source: mSystems. 2018 Mar 13;3(3):e00134-17. doi: 10.1128/mSystems.00134-17 (PMC5864416; doi:10.1128/mSystems.00134-17)
Supplement: TABLE S3 [file sys001182193st3.docx]

| **Sow treatment** | **Control^2^** | | |  | **FMTP^3^** | | |  | **S.E.M** | **P-value** | | |
| --- | --- | --- | --- | --- | --- | --- | --- | --- | --- | --- | --- | --- |
| **Offspring treatment** | **Control^4^** | **FMT1^5^** | **FMT4^6^** |  | **Control** | **FMT1** | **FMT4** |  |  | **Interaction** | **Sow** | **Offspring** |
| White blood cells (x 10^3^ cells/µL) | 26 | 36 | 27 |  | 29 | 27 | 30 |  | 3.4 | 0.12 | 0.76 | 0.39 |
| Lymphocytes |  |  |  |  |  |  |  |  |  |  |  |  |
| % | 27 | 23 | 31 |  | 27 | 26 | 27 |  | 2.2 | 0.21 | 0.95 | 0.13 |
| no. x 10^3^ cells/µL | 6.87 | 7.28 | 8.16 |  | 7.57 | 7.14 | 7.72 |  | 0.498 | 0.49 | 0.93 | 0.25 |
| Monocytes |  |  |  |  |  |  |  |  |  |  |  |  |
| % | 2.1 | 2.4 | 3.0 |  | 3.0 | 2.5 | 2.4 |  | 0.42 | 0.17 | 0.71 | 0.83 |
| no. x 10^3^ cells/µL | 0.52^b^ | 0.78^a,b^ | 0.77^a,b^ |  | 0.83^a^ | 0.66^a,b^ | 0.68^a,b^ |  | 0.083 | 0.02 | 0.59 | 0.79 |
| Granulocytes |  |  |  |  |  |  |  |  |  |  |  |  |
| % | 70 | 61 | 65 |  | 72 | 73 | 61 |  | 7.3 | 0.52 | 0.62 | 0.49 |
| no. x 10^3^ cells/µL | 18.4 | 18.7 | 17.9 |  | 20.5 | 19.2 | 20.1 |  | 2.09 | 0.92 | 0.36 | 0.97 |
| Red blood cells (x 10^6^ cells/µL) | 7.43 | 7.45 | 6.92 |  | 7.44 | 7.53 | 7.27 |  | 0.219 | 0.71 | 0.41 | 0.17 |
| Red cell distribution width (fL) | 19.4^a,b^ | 20.4^a^ | 18.5^b^ |  | 19.4^a,b^ | 18.7^b^ | 19.1^a,b^ |  | 0.44 | 0.04 | 0.37 | 0.19 |
| Haemoglobin (g/dL) | 13.7 | 13.8 | 13.8 |  | 13.5 | 13.9 | 13.4 |  | 0.39 | 0.83 | 0.54 | 0.68 |
| Haematocrit (%) | 0.43 | 0.45 | 0.41 |  | 0.42 | 0.44 | 0.41 |  | 0.015 | 0.92 | 0.59 | 0.18 |
| Mean corpuscular volume (fL) | 58.1 | 59.9 | 58.9 |  | 56.4 | 58.2 | 56.9 |  | 1.08 | 0.98 | 0.05 | 0.25 |
| Mean corpuscular haemoglobin |  |  |  |  |  |  |  |  |  |  |  |  |
| % | 18.5 | 18.6 | 18.5 |  | 18.2 | 18.6 | 18.4 |  | 0.40 | 0.91 | 0.62 | 0.83 |
| Pg | 31.9 | 31.2 | 31.4 |  | 32.2 | 31.9 | 32.4 |  | 0.55 | 0.81 | 0.14 | 0.62 |
| Platelets (x 10^6^ cells /µL) | 288 | 199 | 199 |  | 212 | 191 | 209 |  | 32.5 | 0.39 | 0.36 | 0.21 |
| Mean platelet volume (fL) | 10.03 | 10.02 | 9.65 |  | 9.07 | 9.32 | 9.33 |  | 0.327 | 0.60 | 0.02 | 0.87 |
| Total protein (g/L) | 69.6 | 67.1 | 64.9 |  | 69.8 | 64.3 | 64.7 |  | 1.94 | 0.71 | 0.58 | 0.04 |
| Blood urea nitrogen (mg/dL) | 19.9 | 17.8 | 24.9 |  | 20.2 | 24.4 | 20.9 |  | 2.85 | 0.19 | 0.69 | 0.61 |
| Glucose (mmol/L) | 5.67 | 6.05 | 6.77 |  | 5.90 | 6.73 | 6.98 |  | 0.532 | 0.88 | 0.40 | 0.14 |
| Triglycerides (mmol/L) | 0.64 | 0.64 | 0.59 |  | 0.72 | 0.61 | 0.64 |  | 0.061 | 0.67 | 0.52 | 0.49 |
| Cholesterol (mmol/L) | 2.83 | 2.89 | 3.53 |  | 2.71 | 2.47 | 2.56 |  | 0.295 | 0.37 | 0.04 | 0.44 |
| Creatinine (µmol/L) | 150 | 141 | 151 |  | 157 | 137 | 143 |  | 9.74 | 0.71 | 0.81 | 0.36 |
| Creatine kinase (µmol/L) | 159 | 107 | 158 |  | 176 | 200 | 144 |  | 30.42 | 0.22 | 0.21 | 0.84 |

^1^Least squares means and pooled standard errors of the mean are presented.

Sows were assigned to one of two treatment groups: ^2^Control (Control; n=9) and ^3^FMT procedure (FMTP; n=9); FMTP sows received FMT via gastric intubation on days 70 and 100 of gestation. Piglets were assigned to one of three treatment groups at birth: ^4^Control; ^5^FMT1 (FMT at birth) and ^6^FMT4 (FMT at birth and days 3, 7 and 28 of age).

Data from 36 pigs: Sow treatment level control n=18; FMTP n=18; Offspring treatment level control n=12; FMT1 n=12; FMT4 n=12.

^a,b,c^ Within each row, values that do not share a common superscript are significantly different (P≤0.05).
