# Supplementary material for: Fecal Microbiota Transplantation in Gestating Sows and Neonatal Offspring Alters Lifetime Intestinal Microbiota and Growth in Offspring
Source: mSystems. 2018 Mar 13;3(3):e00134-17. doi: 10.1128/mSystems.00134-17 (PMC5864416; doi:10.1128/mSystems.00134-17)
Supplement: TABLE S4 [file sys001182193st4.docx]

**Table S4**

| **Diet type** | **Starter** | **Link** | **Weaner** | **Finisher** | **Pregnant** | **Lactation** |
| --- | --- | --- | --- | --- | --- | --- |
| Barley | 0.0 | 0.0 | 24.8 | 38.5 | 89.7 | 34.9 |
| Wheat | 22.2 | 39.9 | 43.1 | 40.4 | 0.0 | 43.2 |
| Maize | 8.0 | 0.0 | 0.0 | 0.0 | 0.0 | 0.0 |
| Soya | 16.9 | 22.9 | 20.0 | 17.5 | 7.0 | 15.0 |
| Full fat soya | 10.0 | 7.0 | 5.0 | 0.0 | 0.0 | 0.0 |
| Lactofeed 70^1^ | 20.0 | 20.0 | 0.0 | 0.0 | 0.0 | 0.0 |
| Skim milk powder | 12.5 | 5.0 | 0.0 | 0.0 | 0.0 | 0.0 |
| Soya oil | 7.5 | 2.5 | 4.0 | 10.0 | 10.0 | 40.0 |
| Lysine HCl (78.8) | 0.5 | 0.4 | 0.5 | 4.0 | 1.0 | 3.5 |
| DL-Methionine | 0.3 | 0.2 | 0.2 | 1.0 | 1.0 | 1.0 |
| L-Threonine (98) | 0.2 | 0.2 | 0.2 | 1.5 | 0.0 | 1.0 |
| L-Tryptophan | 0.09 | 0.05 | 0.02 | 0.0 | 0.0 | 0.0 |
| Vitamin and mineral mix | 0.3^2^ | 0.3^2^ | 0.3^2^ | 1.0^3^ | 1.5^4^ | 1.5^4^ |
| Natuphos 5000 FTU/g^5^ | 0.01 | 0.01 | 0.01 | 0.1 | 0.1 | 0.1 |
| Salt feed grade | 0.3 | 0.3 | 0.3 | 3.0 | 4.0 | 4.0 |
| Dicalcium phosphate | 0.1 | 0.1 | 0.6 | 2.0 | 5.0 | 5.0 |
| Limestone flour | 1.1 | 1.1 | 0.9 | 13.0 | 11.0 | 12.0 |
| **Chemical analysis (g/kg dry matter)** | | | | | | |
| Crude protein | 212 | 205 | 180 | 161 | 119 | 148 |
| Crude fibre | 17 | 18 | 25 | 24 | 32 | 25 |
| Crude ash | 56 | 55 | 44 | 41 | 37 | 41 |
| Ether extract | 53 | 52 | 58 | 27 | 29 | 63 |
| Digestible energy (MJ/kg)^6^ | 17.1 | 17 | 16.9 | 16.3 | 15.6 | 16.8 |
| Net energy (MJ/kg)^6^ | 11.4 | 10.3 | 10.6 | 9.8 | 9.5 | 10.5 |
| **Amino acids (g/kg)** | | | | | | |
| Lysine | 15.60 | 14.00 | 13.00 | 11.50 | 6.50 | 9.90 |
| Methionine | 5.50 | 5.40 | 4.20 | 3.70 | 2.00 | 3.40 |
| Methionine + cysteine | 9.50 | 9.20 | 8.10 | 7.40 | 5.00 | 6.80 |
| Threonine | 10.10 | 9.20 | 8.70 | 7.90 | 4.60 | 6.70 |
| Tryptophan | 2.10 | 2.60 | 1.80 | 1.50 | 1.20 | 1.40 |

^1^Lactofeed 70 contains 70% lactose, 11.5% protein, 0.5% oil, 7.5% ash and 0.5% fibre (Volac, Cambridge, UK)*.*

^2^Premix provided per kg of complete diet: Cu, 155 mg; Fe, 90 mg; Mn, 47 mg; Zn, 120 mg; I, 0.6 mg; Se, 0.3 mg; vitamin A, 6000 IU; vitamin D_3,_ 1000 IU; vitamin E, 100 IU; vitamin K, 4 mg; vitamin B_12,_ 15 μg; riboflavin, 2 mg; nicotinic acid, 12 mg; pantothenic acid, 10 mg; choline chloride, 250 mg; vitamin B_1,_ 2 mg; vitamin B_6,_ 3 mg; Endox, 60 g.

^3^Premix provided per kg of complete diet: Cu, 15 mg; Fe, 24 mg; Mn, 31 mg; Zn, 80 mg; I, 0.3 mg; Se, 0.2 mg; vitamin A, 2000 IU; vitamin D_3,_ 500 IU; vitamin E, 40 IU; vitamin K, 4 mg; vitamin B_12,_ 15 μg; riboflavin, 2 mg; nicotinic acid, 12 mg; pantothenic acid, 10 mg; vitamin B_1,_ 2 mg; vitamin B_6,_ 3 mg.

^4^Premix provided per kg of complete diet: Cu, 15 mg; Fe, 70 mg; Mn, 62 mg; Zn, 80 mg; I, 0.6 mg; Se, 0.2 mg; vitamin A, 1000 IU; vitamin D_3,_ 1000 IU; vitamin E, 100 IU; vitamin K, 2 mg; vitamin B_12,_ 15 μg; riboflavin, 5 mg; nicotinic acid, 12 mg; pantothenic acid, 10 mg; choline chloride, 500 mg; biotin, 200 mg; folic acid, 5 g; vitamin B_1,_ 2 mg; vitamin B_6,_ 3 mg.

^5^Phytase; 5000 FTU/g equal to 500 FTU per kg finished feed.

^6^Digestible energy and net energy were calculated from book values.
