# Supplementary material for: Fecal Microbiota Transplantation in Gestating Sows and Neonatal Offspring Alters Lifetime Intestinal Microbiota and Growth in Offspring
Source: mSystems. 2018 Mar 13;3(3):e00134-17. doi: 10.1128/mSystems.00134-17 (PMC5864416; doi:10.1128/mSystems.00134-17)
Supplement: TABLE S5 [file sys001182193st5.docx]

**Table S5**

| **Gene^1^** | **Accession number^2^** | **Gene name** | **Forward primer (5'-3')** | **Reverse primer (5'-3')** | **Amplicon size (bp)** | **Ref^3^** | **Eff. (%)^4^** | **Corr. ^5^** |
| --- | --- | --- | --- | --- | --- | --- | --- | --- |
| *ACTB* | XM_003357928.2 | Beta-actin | GGGCATCCTGACCCTCAAG | TGTAGAAGGTGTGATGCCAGATCT | 89 | 1 | 97.3 | 0.99 |
| *B2M* | NM_213978.1 | Beta-2-microglobulin | CCCCCGAAGGTTCAGGTT | GCAGTTCAGGTAATTTGGCTTTC | 66 | 1 | 102.2 | 0.99 |
| *GAPDH* | NM_001206359.1 | Glyceraldehyde-3-phosphate dehydrogenase | GGCGTGAACCATGAGAAGTATG | GGTGCAGGAGGCATTGCT | 60 | 1 | 96.5 | 0.99 |
| *HPRT1* | NM_001032376.2 | Hypoxanthine guanine phosphoribosyl transferase | AGAAAAGTAAGCAGTCAGTTTCATATCAGT | ATCTGAACAAGAGAGAAAATACAGTCAATAG | 131 | 1 | 92.1 | 0.99 |
| *OAZ1* | NM_001122994.2 | Ornithine decarboxylase antizyme 1 | TCGGCTGAATGTAACAGAGGAA | GAGCCTGGATTGGACGTTTAAA | 70 | 1 | 99.2 | 0.99 |
| *OCLN* | NM_001163647.2 | Occludin | TTGTGGGACAAGGAACGTATTTA | TGCCTGCCGACACGTTT | 76 | 1 | 95.4 | 0.98 |
| *ZO1* | XM_013993251.1 | Zona occludin 1 | AAGCCCTAAGTTCAATCACAATCT | ATCAAACTCAGGAGGCGGC | 131 | 1 | 109.2 | 0.98 |
| *SGLT1 (SLC5A1)* | NM_001164021.1 | Sodium-dependent glucose transporter 1 | TGTCTTCCTCATGGTGCCAA | AGGAGGGTCTCAGGCCAAA | 149 | 1 | 108.0 | 0.99 |
| *GLUT2 (SLC2A2)* | NM_001097417.1 | Facilitated glucose transporter member 2 | TACGGCATCTGCTAGCCTCAT | CCACCAATTGCAAAGATGGAC | 66 | 2 | 89.3 | 1.00 |
| *MCT1 (SLC16A1)* | AM286425.1 | Monocarboxylate transporter 1 | GGTGGAGGTCCTATCAGCAG | AAGCAGCCGCCAATAATCAT | 74 | 1 | 96.4 | 1.00 |
| *SMCT (SLC5A12)* | XM_003122908.1 | Sodium-coupled monocarboxylate cotransporter | AGGTCTACCGCTTTGGAGCAT | GAGCTCTGATGTGAAGATGATGACA | 77 | 2 | 82.3 | 0.99 |
| *GIP* | NM_001287408.1 | Glucose-dependent insulinotropic peptide | GGATGGTGGAGCAGTTGGA | CCAATCCTGAGCTGGGTTTG | 71 | 2 | 98.1 | 0.99 |
| *GLP1* | NM_001256594.1 | Glucagon-like peptide-1 | GCTGATGGTGGCGATCTTGT | TCCCAGCTCTTCCGAAACTC | 69 | 2 | 98.1 | 0.99 |
| *TRL2* | NM_213761.1 | Toll-like receptor 2 | AATAAGTTGAAGACGCTCCCAGAT | GTTGCTCCTTAGAGAAAGTATTGATCGT | 97 | 1 | 92.7 | 0.99 |
| *TRL4* | AB188301.2 | Toll-like receptor 4 | TGTGGCCATCGCTGCTAAC | GGTCTGGGCAATCTCATACTCA | 124 | 1 | 105.8 | 0.98 |
| *ALPI* | XM_003133729.3 | Intestinal alkaline phosphatase | AGGAACCCAGAGGGACCATTC | CACAGTGGCTGAGGGACTTAGG | 83 | 2 | 97.1 | 0.99 |

^1^Gene: Abbreviation with alternate gene names shown in brackets where relevant.

^2^Accession number: National Center for Biotechnology Information (NCBI) Entrez Gene (http://www.ncbi.nlm.nih.gov/sites/entrez?db=gene).

^3^Ref: references for oligonucleotide primer sequences. 1) Metzler-Zebeli BU, Mann E, Ertl R, Schmitz-Esser S, Wagner M, Klein D, Ritzmann M, Zebeli Q. Dietary calcium concentration and cereals differentially affect mineral balance and tight junction proteins expression in jejunum of weaned pigs. Br J Nutr. 2015; 113(7):1019-31. 2) Metzler-Zebeli BU, Ertl R, Grüll D, Molnar T, Zebeli Q. Enzymatically modified starch up-regulates expression of incretins and sodium-coupled monocarboxylate transporter in jejunum of growing pigs. Animal 2016; 11(7):1180-1188.

^4^Eff: PCR efficiency: E = 10^(-1/slope)^-1.

^5^Corr: Correlation coefficient of standard curve.
